# Cancer Stem Cells in Colorectal Cancer: From Molecular Mechanisms to Diagnosis, Prognosis and Therapy Implications

**DOI:** 10.3390/cancers18162569

**Published:** 2026-08-10

**Authors:** Dami Aiyejuto, Ananya Luthria, Thompson Hui, Anitha Kota Shenoy

**Affiliations:** 1 Naresh K. Vashisht College of Medicine, Texas A&M University, Bryan, TX 77807, USA; oaiyejuto7@tamu.edu (D.A.); ananyaluthria6@tamu.edu (A.L.); thui@tamu.edu (T.H.); 2 Department of Medical Education, Naresh K. Vashisht College of Medicine, Texas A&M University, Bryan, TX 77807, USA

**Keywords:** colorectal cancer (CRC), colorectal cancer stem cells (CCSC), cancer stem cells (CSC), tumor microenvironment (TME), therapeutic resistance, metastasis, signaling pathways, diagnostic biomarkers, prognostic biomarkers, therapeutic targeting, clinical trials, drug resistance

## Abstract

Colorectal cancer (CRC) remains a major cause of cancer-related mortality, with recurrence and treatment resistance as persistent challenges. Increasing evidence supports the cancer stem cell (CSC) model in CRC, in which a subpopulation of self-renewing colorectal cancer stem cells (CCSCs) sustains tumor growth, metastasis, and therapy resistance. CCSCs are increasingly recognized as key drivers of tumor progression, therapeutic resistance, and relapse, and are increasingly being investigated not only as therapeutic targets but also as biomarkers with diagnostic and prognostic relevance in CRC. Multiple signaling pathways regulate CCSC maintenance and aggressive behavior, including Wnt/β-catenin, Notch, Hedgehog, PI3K/AKT, MAPK/ERK, NF-κB, and TGF-β signaling. These pathways form the basis of therapeutic strategies aimed at modulating stemness-associated pathways, with several CSC-focused agents and pathway inhibitors currently under translational and clinical investigation. In parallel, CCSC surface markers and molecular signatures are being explored as putative tools for early detection, minimal residual disease monitoring, and outcome prediction. However, successful translation of CSC-directed approaches into effective and safe clinical applications remains difficult. Tumor heterogeneity, CSC plasticity, compensatory signaling mechanisms, and the shared regulatory networks between malignant and normal intestinal stem cells pose substantial barriers. In this review, we summarize the molecular mechanisms underlying CCSC biology, discuss their diagnostic and prognostic implications. We also focus on clinical trials that apply CCSC-directed therapeutic, biomarker, and prevention strategies in CRC., Finally, we emphasize the lessons learned and the translational challenges, including the need for rigorously validated, CCSC-specific biomarkers, that continue to shape the development of these therapeutic and biomarker strategies.

## Simple Summary

Colorectal cancer is often driven by a small population of colorectal cancer stem cells (CCSCs) that fuel tumor growth, metastasis, and treatment resistance, leading to relapse. These CCSCs are regulated by key signaling pathways, including Wnt/β catenin, Notch, Hedgehog, PI3K/AKT, MAPK/ERK, NF-κB, and TGF β, which are now being targeted by emerging drugs. CCSC markers and gene signatures are also under study as diagnostic and prognostic tools for early detection, minimal residual disease monitoring, and outcome prediction, although robust CCSC-specific assays and prospective clinical validation are still lacking. However, translating CCSC directed therapies into safe and effective treatments is difficult due to tumor heterogeneity, CCSC plasticity, compensatory pathways, and overlap with normal intestinal stem cell regulation. This review outlines CCSC biology, their biomarker potential, and current clinical trials targeting CCSC associated pathways, while highlighting major translational barriers that must be overcome.

## 1. Introduction

Colorectal cancer (CRC) is one of the most prevalent malignancies worldwide and remains a leading cause of cancer-related mortality. According to the Global Cancer Observatory, approximately 1.93 million new cases and over 900,000 deaths were attributed to CRC globally in 2020, establishing it as the third most commonly diagnosed cancer and the second leading cause of cancer death [1]. In the United States alone, over 150,000 new cases and more than 53,000 deaths were estimated for 2024 and incidence continues to increase in adults younger than 50 years [2]. These epidemiological patterns underscore the continued global health burden of CRC and the need to better understand the mechanisms that drive progression, recurrence, and treatment failure.

Current treatment strategies for CRC include surgical resection, systemic chemotherapy, radiotherapy, targeted therapy, and immunotherapy [3]. Standard adjuvant chemotherapy regimens are based on fluoropyrimidine agents such as 5-fluorouracil, and the addition of oxaliplatin to fluorouracil and leucovorin has been established as a standard of care following the MOSAIC trial, which demonstrated significant improvement in disease-free survival for patients with stage II and III colon cancer [4]. For patients with microsatellite instability-high or mismatch repair-deficient tumors, immune checkpoint inhibitors such as pembrolizumab have demonstrated significant clinical benefit [5]. Despite these advances, drug resistance remains a major obstacle to long-term treatment success. Disease relapse, metastatic progression, and persistence of minimal residual disease continue to limit durable responses, indicating that conventional treatment approaches may fail to eradicate biologically aggressive tumor cell populations [2,4,5].

Accumulating evidence indicates that a subpopulation of tumor cells with stem cell-like properties, termed cancer stem cells (CSC), plays a central role in mediating therapeutic resistance through mechanisms including autocrine cytokine-mediated apoptosis evasion [6] and microenvironment-driven priming of drug-resistant phenotypes [7]. In CRC, these cells are referred to as colorectal cancer stem cells (CCSCs) and are increasingly recognized as key drivers of tumor initiation, progression, metastasis, and relapse [8].

In this review, we provide a comprehensive overview of the current understanding of CCSC biology in CRC. We discuss the TME and its role in CCSC maintenance, examine CCSC-associated markers and their diagnostic and prognostic significance, and detail the molecular signaling pathways and epigenetic mechanisms that regulate CCSC function. We further explore the role of CCSCs in tumor initiation, progression, metastasis, and therapy resistance. Finally, we review CCSC-relevant clinical trials and emerging technologies and discuss translational challenges and future directions for CCSC-directed therapy in CRC.

## 2. Cancer Stem Cell Concept in CRC

The CSC hypothesis proposes that tumor growth is sustained by a subpopulation of cells possessing the capacity for self-renewal, multilineage differentiation, tumor initiation, maintenance, metastasis and therapeutic resistance.

### 2.1. Historical Evolution of the CSC Concept and Its Extension to CRC

**Figure 1:**
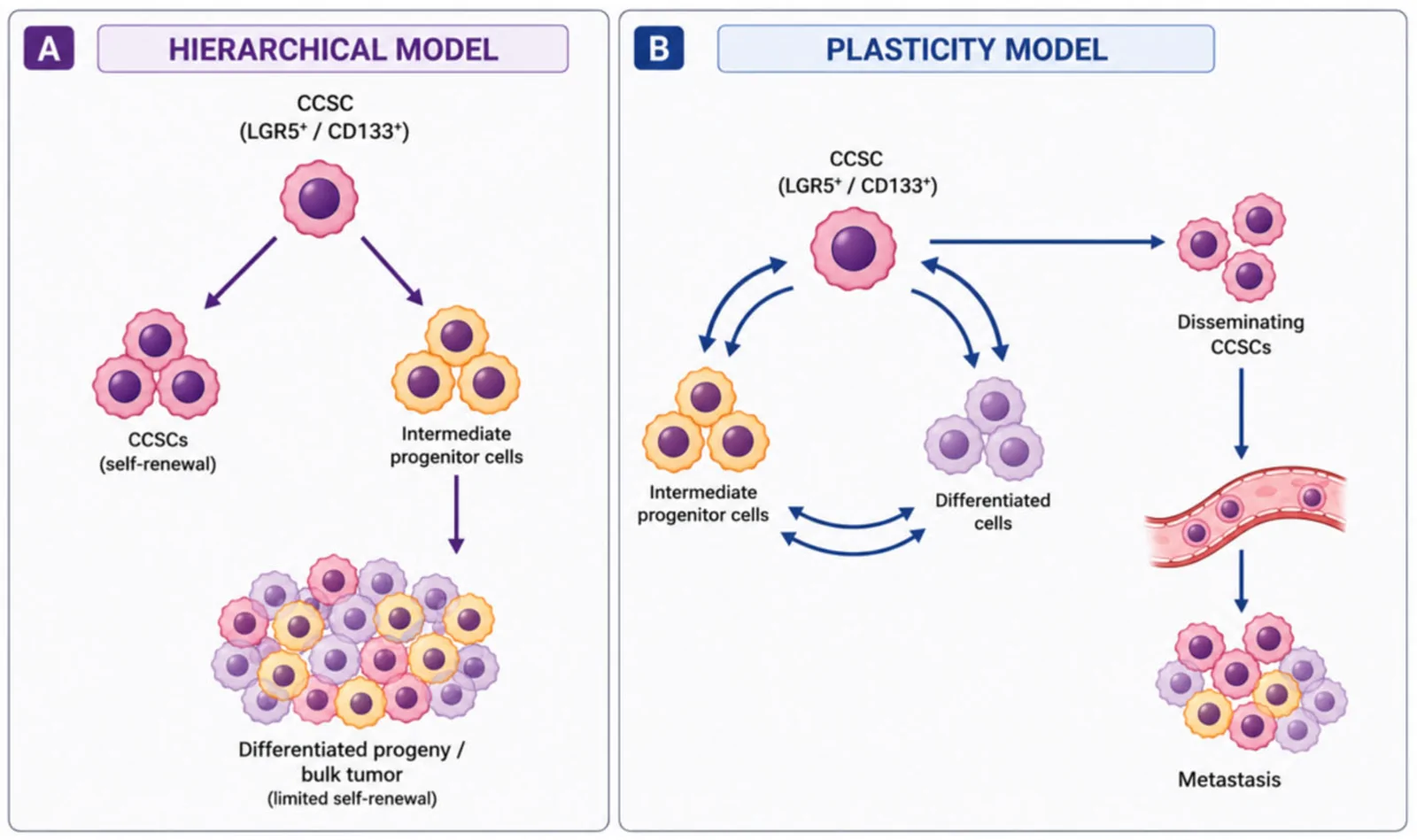
Hierarchical vs. Plasticity Model. (**A**). Hierarchical model: Unidirectional differentiation of CCSCs into progressively differentiated tumor cells. (**B**). Plasticity model: Bidirectional interconversion between CCSCs and differentiated tumor cell states, highlighting cellular plasticity. Shenoy, A. (2026) https://BioRender.com/6f4jnaw.

The modern CSC concept emerged from work in acute myeloid leukemia, where only rare CD34^+^CD38^−^ cells could serially propagate disease in xenograft models, establishing the idea of a functionally defined, self-renewing “tumor-initiating” population [9]. Subsequent demonstration of tumor-initiating cells in solid malignancies, including breast and brain cancers, generalized this model and set the stage for identifying CSCs in epithelial tumors such as CRC [10,11]. In CRC, the existence of CSC was first demonstrated in two landmark studies published in 2007. O’Brien et al. identified a CD133-positive subpopulation of human colon cancer cells capable of initiating tumor growth upon transplantation into immunodeficient mice, providing the first functional evidence for a colon cancer-initiating cell [12]. Concurrently, Ricci-Vitiani et al. confirmed that tumorigenic potential in colon cancer was restricted to the CD133-positive fraction, which accounted for approximately 2.5% of tumor cells and could be serially propagated to reproduce the original tumor phenotype [13]. These observations shifted the view of CRC from a purely stochastic clonal expansion model to one that incorporates hierarchical organization and functional heterogeneity within the tumor cell population. Subsequent studies further refined this concept, leading to two complementary models that explain the origin, maintenance, and dynamic behavior of CCSCs (Figure 1).

### 2.2. Conceptual Models of CCSCs: Hierarchy Versus Plasticity

Two complementary models are used to describe CCSCs (Figure 1). The classical hierarchical CSC model posits a stable stem-cell compartment at the apex of a tumor hierarchy, in which a minority of LGR5^+^ or CD133^+^ CCSCs continuously generate more differentiated progeny with limited self-renewal that form the bulk of the tumor [14]. In this framework, only the apex population can efficiently recapitulate the original tumor in serial xenotransplantation or organoid assays, and durable control requires depletion of these highly tumorigenic cells, as was originally described for CD34^+^CD38^−^ leukemic stem cells in human acute myeloid leukemia [15].

In contrast, the plasticity-based CSC model emphasizes that stem-like and non-stem states are interconvertible and that CCSC properties can be gained or lost in response to microenvironmental cues, therapy, or injury. Lineage-tracing, organoid, and metastasis studies in CRC show that LGR5^−^ or more differentiated cells can reacquire stem-like traits, that disseminating non-CSC cells frequently seed metastases, and that efficient metastatic outgrowth depends on subsequent conversion to CSC states at the secondary site [16]. Recent work, including patient-derived organoid and in vivo plasticity studies, has therefore shifted the CRC stem-cell paradigm from a purely fixed hierarchy toward a dynamic model in which plasticity is a key driver of metastasis and therapeutic resistance, while hierarchical organization still describes the overall epithelial structure [17].

These two models are not mutually exclusive and likely coexist within individual colorectal tumors, with a relatively stable CCSC hierarchy embedded in a broader plastic system that allows non-stem cells to reacquire stemness under selective pressures [18]. From a therapeutic standpoint, strategies aimed solely at depleting apex LGR5^+^ or CD133^+^ CCSCs may debulk the hierarchical compartment but leave open routes for plastic reconstitution of stem-like cells from more differentiated progeny following chemotherapy, targeted therapy, or microenvironmental change. Conversely, interventions that only block state transitions or niche-driven reprogramming may reduce the emergence of new CCSCs without adequately addressing long-lived, pre-existing stem-like clones. Effective CCSC-directed approaches in CRC will therefore need to consider both models simultaneously, targeting established CSC hierarchies and the plasticity mechanisms that sustain or regenerate CCSC populations over time.

### 2.3. Cellular Origins and Relationship to Normal Intestinal Stem Cells

Normal intestinal homeostasis is sustained by LGR5^+^ crypt base columnar stem cells that reside at the bottom of colonic crypts and fuel continuous epithelial turnover through asymmetric division and multipotent differentiation [19,20]. APC loss and consequent Wnt pathway activation in long-lived LGR5^+^ stem cells efficiently initiate adenomas and invasive CRC, supporting a stem‑cell‑of‑origin model [21]. However, as discussed before, accumulating evidence indicates that committed progenitors or even differentiated intestinal cells can reacquire stemness under oncogenic or microenvironmental pressures, underlining the plasticity of epithelial hierarchies and implying multiple potential cells of origin for CCSCs [22,23,24,25]. Recent work using region-specific markers such as NOX1 and NPY1R has further refined this concept by showing that distinct stem cell populations in proximal versus distal colon segments may differentially serve as origins for anatomically and molecularly distinct CRC subtypes [26].

## 3. CCSC Markers

The identification and characterization of CCSCs depends on the use of cell surface markers and functional assays that distinguish tumor-initiating cells (TIC) from the bulk tumor population. Over the past two decades, multiple markers have been proposed for the isolation and enrichment of CCSCs, including CD133, LGR5, CD44, aldehyde dehydrogenase 1 (ALDH1), and epithelial cell adhesion molecule (EpCAM) [27,28,29,30]. Thus, throughout this review, we refer to these markers and related panels as CCSC-enriching markers, recognizing that CCSCs are operationally defined by functional criteria rather than by any single surface antigen. A summary of the detection methods, functional evidence, prognostic significance, major limitations, and current clinical applicability of these markers is provided in Table 1.

### 3.1. CD133 (Prominin-1)

CD133 (Prominin-1) is a pentaspan transmembrane glycoprotein that was the first marker used to prospectively isolate CCSCs from primary human colorectal tumors. In two landmark studies published simultaneously in 2007, O’Brien et al. and Ricci-Vitiani et al. independently demonstrated that CD133-positive cells, which comprised approximately 2.5% of total tumor cells, possessed the exclusive capacity to initiate tumor growth in immunodeficient mice [12,13]. These CD133-positive cells exhibited self-renewal capacity, could be serially transplanted, and reproduced the phenotypic heterogeneity of the parental tumor, providing the first functional evidence for a hierarchically organized CSC population in CRC [12,13]. Todaro et al. further confirmed that CD133-positive colon cancer cells grow as undifferentiated tumor spheroids in vitro and demonstrated that these cells resist apoptosis through autocrine production of interleukin-4 [6]. Experimentally, CD133 is most commonly detected by immunohistochemistry or flow cytometry using AC133 epitope-specific antibodies [12,13].

However, the reliability of CD133 as an exclusive CCSC marker has been challenged. Shmelkov et al. generated a CD133-lacZ knock-in reporter mouse and discovered that CD133 is not restricted to stem cells but is ubiquitously expressed on differentiated colonic epithelium in both mice and humans [31]. In metastatic colon cancers, both CD133-positive and CD133-negative populations were capable of initiating tumors in serial xenotransplantation assays, with the CD133-negative fraction forming more aggressive tumors and expressing the alternative CSC marker CD44 [31]. These findings demonstrated that CD133 expression alone is insufficient to identify the full spectrum of TIC, and that the marker’s utility may be context-dependent, varying between primary and metastatic disease. Taken together, the xenograft and reporter mouse data indicate that CD133 enriches for tumor-initiating activity in some settings but fails to delineate a unique CCSC compartment, limiting its value as a standalone marker and raising concerns about biological heterogeneity across studies. Despite these limitations, CD133 expression has been associated with shorter survival in CRC patients in multiple clinical studies, and it remains one of the most widely studied markers in CCSC research [32]. Although CD133 continues to be extensively investigated as a research marker and is sometimes incorporated into exploratory prognostic panels, it has not been adopted as a standalone clinical biomarker because of its limited specificity and inconsistent expression across tumor populations.

### 3.2. LGR5

Leucine-rich repeat-containing G-protein-coupled receptor 5 (LGR5) was identified as a marker of intestinal stem cells by Barker et al. in 2007 through lineage-tracing experiments in mice [33]. Using knock-in reporter alleles, they demonstrated that LGR5 is exclusively expressed in cycling columnar cells at the crypt base, and that these LGR5-positive cells are capable of long-term self-renewal and multilineage differentiation, generating all epithelial cell types of the small intestine and colon over a 60-day observation period [33]. LGR5 is a Wnt target gene, and its expression is directly dependent on active Wnt/β-catenin signaling, establishing a molecular link between LGR5-positive stem cells and the signaling pathway most frequently dysregulated in CRC [33]. Experimental studies have further demonstrated that LGR5-positive CCRC are enriched for CSC-like properties, including enhanced tumor-initiating capacity and self-renewal compared with more differentiated tumor cells [16,21,29].

In CRC, LGR5 expression has been shown to be significantly elevated compared to normal mucosa, and its overexpression correlates with aggressive clinicopathological features. Wu et al. evaluated LGR5 expression by immunohistochemistry in 192 CRC specimens and found that LGR5 positivity was significantly associated with higher histological grade, greater depth of invasion, lymph node metastasis, distant metastasis, and advanced pTNM stage [34]. LGR5-positive patients had significantly shorter survival than LGR5-negative patients, and multivariate analysis confirmed that LGR5 was an independent prognostic factor [34]. However, the prognostic significance of LGR5 is not without controversy, as some studies have reported that LGR5 overexpression correlates with better outcomes, possibly reflecting differences in tumor subtype, staging, or detection methodology [35]. This bidirectional association suggests that LGR5 immunohistochemistry may capture distinct biological states within a plastic CCSC compartment. These range from proliferative but therapeutically targetable stem-like cells to quiescent, therapy-resistant clones. The observed patterns depend on assay sensitivity, scoring cut-offs, and the underlying molecular subtype. Together, these factors caution against uncritical use of LGR5 as a uniform adverse prognostic marker. Despite this debate, LGR5 remains among the most biologically validated markers of both normal intestinal and CCSCs. However, it has not yet been implemented in routine diagnostic pathology because standardized detection methods and scoring criteria continue to evolve. Instead, LGR5 is primarily used as a research marker to study stemness and is being actively investigated as a prognostic biomarker and potential therapeutic target [20].

### 3.3. CD44

CD44 is a transmembrane glycoprotein that functions as a receptor for hyaluronic acid and participates in cell adhesion, migration, and interactions with the extracellular matrix. CD44 expression is commonly assessed by immunohistochemistry or flow cytometry using pan-CD44 or splice variant-specific antibodies, particularly against the v6 isoform (CD44v6) [14]. Du et al. demonstrated the functional importance of CD44 in CRC by showing that as few as 100 CD44-positive cells isolated from patient tumors could initiate xenograft tumor growth in immunodeficient mice, and that a single CD44-positive cell could form a sphere with characteristic stem cell properties in vitro [36]. Importantly, knockdown of CD44 by RNA interference strongly prevented clonal formation and inhibited tumorigenicity, whereas knockdown of CD133 did not exert similar effects, suggesting that CD44 is of greater functional importance for CCSC activity than CD133 [36]. In the same study, CD44 and CD133 did not colocalize within tumor tissue, indicating that these markers identify distinct but potentially overlapping cell populations [36].

Among the multiple splice variants of CD44, CD44v6 has emerged as a particularly relevant marker of metastatic CCSCs. Todaro et al. demonstrated that all clonogenic CCSC express CD44v6, and that this variant is functionally required for their migration and capacity to generate metastatic tumors [37]. Microenvironmental cytokines, including hepatocyte growth factor (HGF), osteopontin, and stromal-derived factor 1α, were shown to upregulate CD44v6 expression in CCSCs through activation of the Wnt/β-catenin pathway [37]. CD44v6-negative progenitor cells lacked metastatic capacity but could acquire it upon cytokine-induced expression of CD44v6, demonstrating that metastatic competence in CCSCs can be dynamically reprogrammed by the microenvironment [29]. In patient cohorts, low CD44v6 expression was associated with improved survival, further supporting its value as both a functional biomarker and a therapeutic target. This conclusion was reinforced by subsequent meta-analytic data linking CD44v6 overexpression to lymph node invasion, distant metastasis, and poorer overall survival in CRC [37]. These prognostic data are not entirely uniform, and some studies have reported that loss or low expression of CD44v6 associates with more aggressive tumor features and poorer outcome [38], whereas others link CD44v6 overexpression to lymph node metastasis, distant spread, and reduced survival [39]. These apparently divergent findings likely reflect differences in patient populations, disease stage, scoring thresholds, tumor compartments analyzed, and treatment settings, and together suggest that CD44v6 expression marks biologically distinct CCSC and tumor states rather than serving as a simple unidirectional prognostic indicator. Nevertheless, CD44 is broadly expressed on inflammatory and stromal cells, and splice-variant heterogeneity complicates standardized detection of CD44v6 [14,37,40]. Consequently, despite its strong functional and prognostic significance, CD44/CD44v6 has not been adopted for routine diagnostic use and is currently investigated primarily as a research marker, prognostic biomarker, and potential therapeutic target [14,40].

### 3.4. ALDH1

Aldehyde dehydrogenase 1 (ALDH1) is an intracellular enzyme involved in the oxidation of intracellular aldehydes and the metabolism of retinol to retinoic acid, and its activity has been used as a functional marker for both normal and malignant stem cells. Huang et al. demonstrated that ALDH1 is a marker of normal and malignant human colonic stem cells and that ALDH1 expression tracks stem cell overpopulation during colon tumorigenesis [41]. Using the ALDEFLUOR assay to isolate cells with high ALDH enzymatic activity, they showed that ALDH1-positive cells were enriched for tumor-initiating capacity and could generate xenograft tumors at limiting dilutions [41]. ALDH1-positive cells were localized to the bottom of normal colonic crypts and expanded progressively during the adenoma-to-carcinoma transition, consistent with the hypothesis that tumorigenesis involves an expansion of the stem cell compartment [41]. In CRC specimens, high ALDH1 expression has been associated with adverse clinicopathologic features and poorer survival, supporting its role as both a functional CCSC marker and a negative prognostic biomarker [42,43,44,45]. Unlike surface markers such as CD133 and CD44, ALDH1 can be evaluated either by measuring enzymatic activity using the ALDEFLUOR assay or by immunohistochemical detection of ALDH1 protein, providing complementary functional and protein-based approaches to CCSC identification [41,44]. However, differences in substrate choice, gating strategies, and cut-offs for ‘ALDH^high^’ cells across studies can yield non-comparable CSC fractions, and the inclusion of normal progenitors and non-CSC ALDH-expressing cells within the ALDEFLUOR-positive gate limits the specificity of ALDH1 as a solitary CCSC identifier. Despite its strong functional relevance, similar to LGR5 and CD44, ALDH1 has not been adopted for routine clinical use and remains primarily a research marker, while continuing to be investigated as a functional CCSC marker and candidate prognostic biomarker in CRC [14].

### 3.5. EpCAM and CD166

Epithelial cell adhesion molecule (EpCAM) is a transmembrane glycoprotein involved in cell–cell adhesion, signaling, and differentiation. EpCAM expression is commonly evaluated by immunohistochemistry and flow cytometry, most often in combination with other CCSC markers [46]. Although EpCAM is broadly expressed on epithelial tissues, its high expression level (EpCAM^high^) has been used in combination with other markers to enrich CCSCs, rather than serving as a CCSC-specific marker on its own. Dalerba et al. performed the first comprehensive phenotypic characterization of CCSCs using multiparameter flow cytometry and demonstrated that in six of six human CRCs tested, tumor-initiating capacity was restricted to an EpCAM^high^/CD44^+^ subpopulation [46]. These cells reproduced the full morphologic and phenotypic heterogeneity of their parental tumors upon xenotransplantation [46]. Further characterization revealed CD166 (activated leukocyte cell adhesion molecule) as an additional differentially expressed marker within the EpCAM^high^/CD44^+^ fraction, enabling further enrichment of CCSCs [46]. Liu et al. subsequently confirmed the clinical significance of the EpCAM^high^/CD44^+^ phenotype, demonstrating that this marker combination was significantly correlated with depth of invasion, clinical stage, and metastatic status in a cohort of 80 CRC patients, and that EpCAM^high^/CD44^+^ were detectable in corresponding liver metastases [47]. Consistent with these findings, elevated EpCAM expression in CRC has been associated with more aggressive clinicopathologic features and poorer outcomes in several cohorts, further supporting its utility as part of CCSC-enriching marker panels [47,48]. Notably, although EpCAM overexpression is frequently associated with adverse clinicopathologic features, reduced membranous EpCAM expression at the invasive tumor front has also been linked to epithelial–mesenchymal transition (EMT), invasion, and disease progression, indicating that its biological significance is context dependent [48,49]. Despite its utility in enriching CCSC populations when combined with other markers, EpCAM has not been adopted as a CCSC-specific clinical biomarker because of its widespread expression in normal epithelial tissues [14,47,50]. Instead, it is used primarily as a component of multiparameter research panels and circulating tumor cell enrichment platforms while continuing to be investigated as a candidate prognostic biomarker in CRC [14,48,50].

### 3.6. Combinatorial Marker Approaches

As individual markers have shown limitations in specificity and consistency across CRC subtypes, combinatorial marker strategies have been developed to improve the identification and enrichment of CCSCs. The initial observation by Dalerba et al. that EpCAM^high^/CD44^+^/CD166^+^ cells more reliably identify tumor-initiating populations than any single marker alone established the precedent for multi-marker panels [46]. Subsequent studies have extended this approach by demonstrating that the triple-positive LGR5^+^/CD44^+^/EpCAM^+^ phenotype more strictly defines CCSC with enhanced self-renewal and tumorigenic capacity compared to cells identified by any individual marker [29]. Lugli et al. evaluated the prognostic impact of five putative CSC markers (CD133, CD166, CD44, EpCAM, and ALDH1) in a large tissue microarray cohort and found that the loss of expression of multiple markers, rather than the overexpression of any single marker, was associated with adverse clinicopathological features and poor survival [49]. This finding underscores the complexity of CCSC phenotypes and supports the use of multi-marker approaches for both biological studies and clinical prognostic stratification.

Taken together, the data on CD133, CD44, ALDH1, and LGR5 indicate that each marker captures only a subset of CCSC states, with substantial context-dependence across primary versus metastatic sites, molecular subtypes, and assay platforms [49,51,52]. Accordingly, CCSC-associated markers should be viewed as tools that enrich for putative CCSC fractions, which must then be functionally validated using transplantation, organoid-forming, and treatment-resistance assays rather than assumed to represent a uniquely defined stem cell compartment [12,13,49,53]. Studies reporting strong prognostic associations often rely on single-marker immunohistochemistry or enzymatic assays that do not fully resolve intratumoral heterogeneity, while reporter-based and functional xenograft approaches reveal overlapping but non-identical CSC compartments, exposing gaps in specificity and reproducibility [54]. These inconsistencies explain why no single marker has achieved routine clinical adoption and argue for more rigorous standardization of detection methods, integration of combinatorial panels with functional readouts, and stratification by molecular subtype in future studies rather than extrapolating universal CCSC signatures from heterogeneous datasets. By explicitly acknowledging these limitations, multi-marker and multimodal strategies can be designed to prioritize biological robustness and translational feasibility over nominal marker novelty. To facilitate direct comparison across markers, Table 1 summarizes for CD133, CD44, LGR5, EpCAM, and ALDH1 the detection methods, key functional and prognostic evidence, major limitations, and current level of clinical applicability.

From a comparative standpoint, CD133, CD44, ALDH1, LGR5, EpCAM, and CD166 each contribute distinct strengths and limitations as CCSC biomarkers [55]. CD133 and CD44 were among the earliest markers used for functional CCSC isolation, but CD133 lacks specificity and is expressed on differentiated epithelium, whereas CD44 (particularly CD44v6) shows stronger functional links to clonogenicity, migration, and metastasis [51]. LGR5 is uniquely validated as a normal intestinal stem-cell marker and correlates with aggressive behavior, yet its prognostic association is bidirectional and may reflect plastic, context-dependent CCSC states [56]. ALDH1 provides a functional, enzymatic readout of stem-like activity but captures heterogeneous ALDH^high^ populations that include non-CSC cells [57]. EpCAM and CD166, while not CCSC-specific, enhance enrichment when used in multi-marker panels such as EpCAM^high^/CD44^+^/CD166^+^ [46,48], highlighting that combinatorial approaches generally outperform single markers for both biological and prognostic applications.

## 4. CCSCs in Tumor Initiation, Progression, and Metastasis

CCSCs are widely recognized as key drivers of colorectal tumor initiation, progression, and metastatic spread, providing a unifying explanation for intratumoral heterogeneity, therapeutic resistance, and disease recurrence [14,28]. Building on the hierarchical model of tumorigenesis, CCSCs are operationally defined by functional assays and are enriched via markers such as CD133, CD44, EpCAM^high^, LGR5, ALDH1, and CD166 [49,52]. In practice, functional validation of putative CCSCs relies on a series of assays that demonstrate self-renewal, tumor-initiating capacity, and durable propagation. These include in vivo limiting-dilution xenotransplantation to quantify tumor-initiating cell frequency, serial transplantation to show long-term self-renewal, lineage-tracing experiments in genetically engineered mouse models, organoid- and sphere-forming assays in vitro, and treatment-resistance assays that compare survival of marker-defined populations after chemotherapy or targeted agents [14,58]. Together, these approaches establish that CCSCs constitute a numerically minor but biologically dominant compartment capable of sustaining colorectal tumors over time. Consistent with these functional criteria, CCSC-enriched fractions have been shown to support long-term self-renewal, multilineage differentiation, and serial tumor propagation at both primary and metastatic sites [28,59]. The tumor-initiating capacity of CCSCs has been functionally established by xenotransplantation experiments showing that only marker-defined subpopulations such as CD133^+^ and EpCAM^high^/CD44^+^ cells can efficiently generate tumors in immunodeficient mice. These cells can be serially transplanted while recapitulating the morphologic and phenotypic heterogeneity of the parental lesions, providing strong functional support for the CCSC model [12]. However, xenograft-based readouts have important limitations, including dependence on immunodeficient hosts, species-specific microenvironmental cues, and incomplete representation of human immune and stromal interactions, underscoring the need to integrate transplantation studies with lineage-tracing and organoid models when defining CCSCs.

Beyond tumor initiation, CCSCs contribute to tumor progression and maintenance through sustained proliferation and resistance to apoptosis. Their ability to self-renew and differentiate enables continuous tumor growth while maintaining cellular diversity within the tumor [60]. These features are driven by aberrant activation of canonical stemness pathways, including Wnt/β-catenin, Notch, Hedgehog, TGF-β/BMP, and PI3K/AKT, together with genetic and epigenetic alterations in key CRC driver genes, which are discussed in detail in a subsequent section of this review.

Importantly, CCSCs play a critical role in metastasis, the primary cause of CRC-related mortality. Studies have shown that cells with high stemness characteristics exhibit increased invasive and migratory capacity. This facilitates dissemination to distant organs such as the liver and lung, which are the predominant metastatic sites in CRC and exemplify the organotropism of CCSCs [14,61,62]. The metastatic process is closely associated with EMT, during which tumor cells acquire stem-like properties and enhanced motility [63]. Emerging data suggest that metastasis-initiating CCSCs often display a hybrid epithelial/mesenchymal phenotype that preserves cell–cell adhesion while conferring motility, thereby supporting collective migration and efficient metastatic seeding [62,63]. CCSCs also contribute to pre-metastatic niche formation via secretion of cytokines, chemokines, and extracellular vesicles (EV) that condition distant tissues, promote vascular leakiness, remodel the extracellular matrix, and recruit bone-marrow-derived cells, particularly in the liver [62,64].

## 5. CCSC and Tumor Microenvironment

CCSCs do not exist in isolation but are embedded within a complex and dynamic tumor microenvironment (TME) that profoundly influences their behavior. The TME is composed of a heterogeneous mixture of non-neoplastic cell types and extracellular components that surround and interact with tumor cells. Key cellular constituents of the TME include cancer-associated fibroblasts (CAFs), tumor-associated macrophages (TAMs), T lymphocytes, endothelial cells, and other immune and inflammatory cell populations [65]. These cellular components are situated within an extracellular matrix (ECM) composed of collagens, glycoproteins, and proteoglycans, and are bathed in a milieu of soluble signaling molecules including cytokines, chemokines, and growth factors. Additionally, physical features of the TME, such as hypoxia, acidic pH, and altered nutrient availability, further shape the behavior of both tumor cells and their surrounding stroma [66]. Together, these elements form a specialized niche that regulates CCSC self-renewal, differentiation, proliferation, therapy resistance, and metastatic potential.

**Figure 2:**
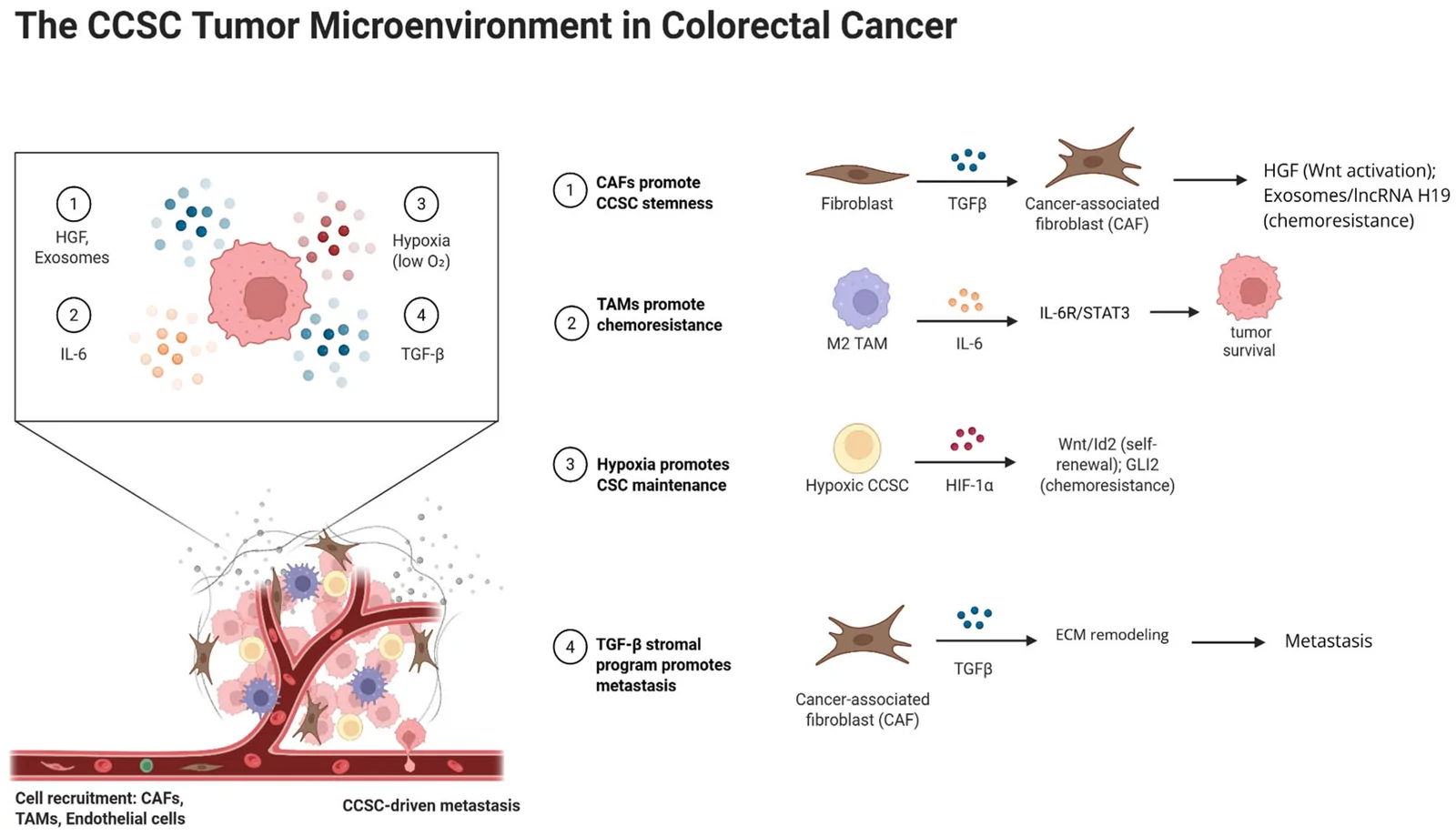
The CCSC Tumor Microenvironment in Colorectal Cancer. Schematic overview of the key components of the TME that regulate CCSC maintenance, chemoresistance, and metastatic progression. (1) Cancer-associated fibroblasts (CAFs) promote CCSC stemness by secreting hepatocyte growth factor (HGF), which enhances Wnt/β-catenin signaling, and by transferring exosomal lncRNA H19, which confers chemoresistance. (2) Tumor-associated macrophages (TAMs), polarized toward an M2 phenotype, secrete interleukin-6 (IL-6), which activates the IL-6R/STAT3 signaling axis in CCSCs, promoting tumor cell survival and resistance to chemotherapy. (3) Hypoxic conditions within the TME stabilize HIF-1α, which maintains CCSC self-renewal through Wnt/β-catenin-dependent reactivation of Id2 and synergizes with TGF-β2 to activate GLI2, promoting intrinsic chemoresistance. (4) TGF-β-driven transcriptional programs in stromal cells activate CAFs to remodel the extracellular matrix (ECM), facilitating tumor cell dissemination and metastatic initiation. Aiyejuto, D. (2026) https://BioRender.com/1x7cmoq.

Among the stromal cell populations within the TME, CAFs are one of the most abundant and functionally significant regulators of CCSC maintenance (Figure 2). In CRC, CAFs have been shown to promote CSC self-renewal and stemness through paracrine signaling. Vermeulen et al. demonstrated that high Wnt activity functionally defines the CCSC population, and that this Wnt activity is not solely determined by intrinsic genetic mutations but is dynamically regulated by the microenvironment [67]. Specifically, stromal myofibroblasts were found to secrete HGF and other factors that enhanced Wnt signaling in adjacent tumor cells, restoring clonogenic capacity even in more differentiated cancer cells with low Wnt activity [67]. This finding established that CCSC identity is plastic and that extrinsic signals from the stroma can actively maintain or restore stem cell-like properties in CRC.

Beyond soluble paracrine factors, CAFs also communicate with CCSCs through the secretion of EV. Ren et al. demonstrated that CAFs transfer the long non-coding RNA H19 to CRC cells via exosomes, which activates Wnt/β-catenin signaling by acting as a competing endogenous RNA sponge for miR-141. This in turn promotes cancer stemness and confers resistance to oxaliplatin [68]. Similarly, Hu et al. showed that fibroblast-derived exosomes prime CCSCs for chemoresistance by increasing the proportion of CD133-positive TIC and enhancing sphere-forming capacity following treatment with 5-fluorouracil or oxaliplatin [7]. These studies reveal that the crosstalk between CAFs and CCSCs extends beyond traditional cytokine-mediated signaling and involves exosome-mediated transfer of regulatory RNAs, representing a sophisticated mechanism by which the TME sustains stemness and drives therapeutic resistance. More generally, EV-mediated transfer of resistance-associated RNAs and proteins from CSCs or CAFs to bulk tumor cells has emerged as a recurrent mechanism of acquired chemoresistance in CRC and other malignancies [69]. Additionally, Deng et al. demonstrated that even at the earliest stages of CRC (T1 tumors), CAFs promote tumor cell invasion through direct cell–cell interactions and stage-specific extracellular matrix remodeling via the cysteine protease cathepsin H, indicating that stromal support of tumor progression begins at the onset of tumorigenesis [70].

Beyond CAF-derived exosomes, CCSC–derived EVs are increasingly recognized as central mediators of CCSC communication with the colorectal TME [71]. CSC-derived EVs in CRC carry miRNAs, lncRNAs, proteins, and metabolites that induce stem-like properties in non-CSC colorectal tumor cells [72]. They also remodel colonic stromal and immune cell function and prime pre-metastatic niches in typical target organs such as liver and lung [73]. Together, these effects contribute to immune evasion, metastatic spread, and resistance to standard CRC chemotherapies. These observations place EVs alongside cytokines and direct cell–cell interactions as a major, and still evolving, route through which CCSCs shape local colonic tumor ecology and distant metastatic sites.

TAMs represent another critical component of the CRC microenvironment that influences CCSC behavior (Figure 2). TAMs are frequently polarized toward an M2-like, immunosuppressive phenotype within the TME, and their infiltration has been associated with poor clinical outcomes in CRC [74]. Yin et al. demonstrated that TAM infiltration is directly associated with chemoresistance in CRC patients, and that CRC-conditioned macrophages confer chemoresistance by secreting interleukin-6 (IL-6), which activates the IL-6R/STAT3 signaling axis in tumor cells [74]. This TAM-derived IL-6 signaling transcriptionally suppresses the tumor suppressor miR-204-5p, thereby promoting cancer cell survival and resistance to drug-induced apoptosis [74]. These findings highlight the immune microenvironment as a key contributor to CCSC-mediated therapy resistance and suggest that immunomodulatory strategies targeting TAM-derived cytokines may enhance the efficacy of conventional chemotherapy.

Hypoxia is a hallmark physical feature of the TME in solid tumors, including CRC, and plays a critical role in maintaining CCSC populations (Figure 2). Regions of low oxygen tension arise because of rapid tumor cell proliferation outpacing the development of functional vasculature. The cellular response to hypoxia is primarily mediated through hypoxia-inducible factors (HIFs), which activate transcriptional programs involved in angiogenesis, metabolic adaptation, and stemness maintenance. In CRC, Dong et al. demonstrated that hypoxia promotes the self-renewal of CCSCs through Wnt/β-catenin signaling-dependent reactivation of Id2 expression, maintaining the hierarchical organization of the CSC population under low-oxygen conditions [75]. Santoyo-Ramos et al. further showed that both HIF-1α and HIF-2α are essential for the tumorigenic capacity of colon cancer cells and modulate the expression of stemness markers such as CD44 and Oct4, although they exert opposing effects on canonical Wnt signaling [18]. Importantly, Tang et al. demonstrated that HIF-1α and CAF-secreted TGF-β2 converge to synergistically activate GLI2, a Hedgehog pathway transcription factor, in CCSCs. This convergence promotes stemness and intrinsic resistance to chemotherapy, and high expression of HIF-1α/TGF-β2/GLI2 was robustly correlated with patient relapse following chemotherapy [76]. These findings reveal that hypoxia does not simply reflect inadequate oxygenation but actively drives CCSC maintenance through complex interactions with both intrinsic signaling pathways and extrinsic stromal cues. This directly connects the CCSC niche to the core signaling networks that underlie CCSC maintenance and plasticity, which are explored in the following section.

The TME also influences CCSC behavior through stromal-derived signaling programs that promote metastatic progression (Figure 2). Calon et al. demonstrated that CRC metastasis depends on a TGF-β-driven transcriptional program in stromal cells, in which TGF-β signaling activates CAFs to produce extracellular matrix components and secreted factors that facilitate tumor cell dissemination [77]. This study established that the paracrine interaction between tumor cells and their surrounding stroma, mediated by TGF-β, is a critical determinant of metastatic initiation in CRC. Notably, this stromal activation by TGF-β was associated with poor prognosis and disease relapse in CRC patient cohorts, further emphasizing the clinical relevance of the TME in shaping disease outcomes [77].

Beyond CAFs, TAMs, and hypoxia, colorectal tumors frequently harbor expanded populations of tumor-associated neutrophils, myeloid-derived suppressor cells (MDSCs), and FOXP3^+^ regulatory T cells, which inhibit cytotoxic lymphocyte activity, promote angiogenesis and metastasis, and have been linked to enhanced cancer stemness and poor prognosis [78].

Given the central role of the TME in CCSC maintenance and therapy resistance, therapeutic strategies that target the microenvironment have emerged as a complementary approach to conventional and CSC-directed therapies. Several avenues of TME-targeted intervention are under investigation. Neutralization of IL-4, which CCSCs produce in an autocrine manner to evade apoptosis, has been shown to sensitize CCSCs to chemotherapy-induced cell death both in vitro and in vivo, demonstrating that disrupting cytokine-mediated survival signaling within the TME can overcome CSC-intrinsic resistance [6]. Targeting TAM-derived IL-6/STAT3 signaling represents another promising approach, as blocking this axis has been shown to reduce chemoresistance in preclinical CRC models [74]. Additionally, inhibition of the TGF-β pathway in stromal cells has been demonstrated to impair metastatic initiation, suggesting that targeting the stromal compartment may prevent disease dissemination [77]. The convergence of HIF-1α and TGF-β2 on GLI2 activation in CCSCs further identifies GLI2 as a potential therapeutic node at the intersection of hypoxia and stromal signaling [76]. Strategies aimed at normalizing tumor vasculature to alleviate hypoxia, disrupting CAF-derived exosomal communication, and modulating immune cell polarization within the TME are also being explored. However, the development of TME-targeted therapies must account for the complexity and context-dependency of TME interactions, as certain stromal components may also exert tumor-suppressive effects under specific conditions.

## 6. Molecular Regulation of CCSCs

As seen in the preceding sections, multiple lines of evidence already point to a central role for signaling pathways in shaping CCSC behavior within their niche. CCSCs are regulated by a complex network of signaling pathways and transcriptional programs that regulate self-renewal, differentiation, tumor initiation, metastasis, and therapeutic resistance [8]. Many of these mechanisms mirror those that govern normal intestinal stem cells in small intestine and colon, including canonical Wnt/β-catenin, Notch, Hedgehog, PI3K/AKT/mTOR, and TGF-β/BMP signaling, which together sustain crypt stem-cell homeostasis and epithelial renewal. Among these, Wnt/β-catenin and LGR5-linked signaling represent particularly dominant axes in both normal intestinal stem cells and CCSCs, making them attractive but high-risk therapeutic targets from the standpoint of on-target toxicity [79].

A comprehensive understanding of these regulatory networks is essential for identifying effective therapeutic strategies aimed at targeting CCSCs, overcoming treatment resistance, and preventing tumor recurrence, while minimizing on-target damage to normal intestinal stem cells and preserving crypt homeostasis.

In parallel with these signaling cascades, colorectal tumors and CCSCs undergo metabolic reprogramming, including enhanced glycolysis (Warburg effect), altered mitochondrial oxidative metabolism, and rewired lipid and amino-acid utilization [80]. These changes provide ATP, biosynthetic precursors, and redox balance under conditions such as hypoxia and nutrient stress, and have been linked to stem-like phenotypes, EMT, and resistance to therapy in CRC models [80,81]. Emerging evidence suggests that CCSC-enriched subpopulations can display distinct metabolic profiles compared with bulk tumor cells, including greater dependence on oxidative phosphorylation and discrete lipid-metabolic circuits [82,83]. These CCSC-biased metabolic features raise the possibility that metabolic enzymes and transporters may serve as CCSC-selective points of intervention when combined with conventional therapies.

### 6.1. Signaling Pathways

Multiple intracellular signaling pathways govern the maintenance, survival, and plasticity of CCSCs. Many of these pathways, including Wnt/β-catenin, Notch, Hedgehog, TGF-β/BMP, NF-κB, and PI3K/AKT/mTOR, are normally involved in intestinal stem cell biology but become aberrantly activated upon the development of CRC thereby driving tumorigenesis, metastasis, and therapeutic resistance.

Rather than acting independently, they form an interconnected network with extensive cross-talk, which helps explain the emergence of treatment-refractory clones and supports the rationale for multi-targeted or combination therapeutic approaches. In the following subsections, we examine the major CCSC-associated signaling cascades and how their dysregulation, together with microenvironmental signals, maintains stem-like traits and drives key malignant processes, including tumor initiation, EMT, angiogenesis, and metastatic spread.

#### 6.1.1. Wnt/β-Catenin

The Wnt/β-catenin pathway is widely considered the central regulator of CCSC maintenance and intestinal stem cell homeostasis. In normal intestinal tissue, Wnt signaling regulates stem cell renewal within the intestinal crypts, maintaining a balance between proliferation and differentiation. Barker et al. elegantly demonstrated this by identifying LGR5-positive crypt base columnar cells as self-renewing stem cells in the small intestine and colon [33]. In CRC, mutations in key components of the pathway, such as Adenomatous polyposis coli (APC), lead to stabilization and nuclear accumulation of β-catenin, which can interact with TCF/LEF transcription factors to activate genes involved in proliferation, survival, and stemness [84]. APC is mutated in more than 80% of CRCs, and sustained Wnt/β-catenin activity is required not only for tumor initiation but also for maintenance of APC-mutant colorectal tumor xenografts, indicating its non-redundant role in tumor persistence [85].

The intestinal stem cell data dovetail with CCSC studies showing that Wnt activity itself functionally marks the tumor-initiating compartment. Vermeulen et al. used a Wnt reporter in human colon cancers and demonstrated that cells with high Wnt activity are enriched for CCSC markers, display increased clonogenicity, and show superior tumor-initiating capacity in xenograft models [67]. In the same study, Wnt^high^ tumor cells localized preferentially near stromal myofibroblasts. Conditioned medium from these myofibroblasts, particularly HGF, enhanced β-catenin–dependent transcription and clonogenicity, and even restored a CCSC phenotype in more differentiated Wnt^low^ cells. Together, these observations demonstrate that microenvironmental signals can reprogram Wnt-dependent CCSC states, reinforcing the central role of this pathway in CCSC maintenance and plasticity [67].

In CCSCs, persistent activation of Wnt signaling promotes tumor initiation, and supports metastatic potential. It also regulates many key CSC markers, including LGR5, further reinforcing its role in maintaining stem-cell-like properties, driving tumor progression and therapeutic resistance [86]. Mechanistically, Wnt target genes involved in stemness and survival such as *MYC*, *AXIN2*, *LGR5* and anti-apoptotic mediators are upregulated in Wnt-active CCSCs [33,87,88,89]. Maintenance of APC-mutant CRC remains critically dependent on Wnt/β-catenin signaling, even after tumors are established [90]. This indicates that the CCSC compartment, and not just early initiating lesions, requires sustained pathway activity for continued growth and survival. Consistent with this, experimental inhibition of Wnt/β-catenin signaling in APC-mutant CRC models result in impaired growth of established tumors, increased differentiation, and reduced tumor-initiating capacity, reflecting functional depletion or exhaustion of CCSCs. In this context, Waaler et al. showed that a novel tankyrase inhibitor, by stabilizing AXIN and enhancing destruction-complex activity, decreased canonical Wnt signaling, reduced growth of conditional APC-mutant colon tumors, and induced a more differentiated phenotype in vivo, consistent with loss of a Wnt-dependent CCSC pool [91]. Moreover, therapeutic perturbations such as MEK inhibition can paradoxically activate Wnt signaling and enrich for LGR5-positive, stem-like CRC cells, providing a direct link between Wnt-driven CCSC plasticity and acquired resistance to targeted therapies [92].

#### 6.1.2. Notch

The Notch signaling pathway plays an important role in intestinal cell fate determination and stem cell maintenance [93,94]. The pathway is activated via binding of Delta-like or Jagged ligands to Notch receptors. Activation of Jagged/Delta ligands induces γ-secretase-mediated release of the Notch intracellular domain (NICD), which associates with RBPJ/CSL and MAML to induce the transcriptional repressors HES1 and HEY1 necessary for proliferation and differentiation [94].

In normal intestinal epithelium, Notch signaling functions to maintain progenitor cell populations and suppress differentiation, particularly via inhibition of secretory lineage commitment. Disruption of the Notch signaling leads to rapid differentiation and loss of proliferative capacity [95].

In CRC, dysregulated Notch signaling contributes to tumor progression, metastasis, and maintenance of CSC populations. Sikandar et al. demonstrated that Notch activity is markedly elevated in colon cancer-initiating cells and is required for their self-renewal and repression of secretory differentiation, directly implicating Notch as a core regulator of CCSC maintenance in established tumors [96]. Studies have shown the mechanistic role of Notch signaling functions in promoting metastatic behavior. It has been shown that ALDH1A1 enhances CRC metastasis via activation of the Notch pathway, linking metabolic enzyme regulation to CSC-associated signaling and tumor dissemination [97]. Similarly, an alternative study demonstrated that epigenetic regulation via demethylated miR-184 suppresses metastasis by downregulating Notch signaling, highlighting the importance of epigenetic control in modulating tumor progression via Notch signaling [98].

In addition, Notch signaling is also important in regulating tumor cell behavior in early metastatic events. A recent study showed that Notch signaling governs the growth and survival of detached CRC cell clusters, suggesting that it plays a role in enabling tumor cells to adapt to anchorage-independent conditions and facilitating dissemination [99]. Importantly, substantial crosstalk exists between the Notch and Wnt signaling pathways, both cooperatively regulating the CCSC function. Activation of both pathways maintains the undifferentiated, self-renewing state of CCSCs, while modulating Notch signaling can alter CSC plasticity, even in the presence of active Wnt signaling [100].

#### 6.1.3. Hedgehog

The Hedgehog (Hh) signaling pathway is a developmental pathway involved in stem cell regulation and tumor progression [101]. Typical Hh signaling is initiated by the binding of Hh ligands (Sonic, Indian, or Desert Hh) to the Patched receptor. This relieves inhibition of Smoothened (SMO) and ultimately activates GLI transcription factors, which regulate genes involved in proliferation, survival, and differentiation [101].

Consistent with the broader CSC model, Hedgehog signaling enhances self-renewal, survival, and metastatic potential of CSCs in multiple solid tumors, supporting its role as a CCSC-modulating pathway and a rational therapeutic target in CRC [102]. In CRC, Hh signaling predominantly operates via paracrine activation of stromal cells, complementing the CAF- and TAM-mediated pathways discussed in the TME section. This stromal activation regulates tumor behavior and contributes to the maintenance of CCSC populations [103]. Yauch et al. first demonstrated in pancreatic and colon cancer models that tumor-derived Sonic Hh activates Hh signaling in stromal cells, and that pharmacologic Hh blockade remodels the stroma and alters tumor growth, supporting a paracrine, microenvironment-mediated role for Hh in sustaining CSC-like cells [104].

Gerling et al. showed that stromal Hh signaling is downregulated in CRC, and that restoration of this signaling suppresses tumor growth and reduces stem-like tumor characteristics [105]. This highlights a tumor-suppressive role of stromal Hh signaling, emphasizing the importance of tumor-stroma interactions in regulating cancer progression. Hh signaling has also been shown in preclinical colon cancer models to directly sustain a stem-like compartment, where autocrine, non-canonical, GLI-independent Hh activity positively regulates Wnt signaling and is required for CCSC survival, illustrating how context-dependent Hh signaling can promote CCSC expansion and chemoresistance rather than restrain CCSC pools [106].

More recent studies have shown that Hh signaling influences the tumor immune microenvironment [107]. The modulation of Hh activity alters cytokine profiles and immune-cell infiltration patterns in colorectal tumors, suggesting a role for Hh signaling in immune regulation and potential immune evasion that may indirectly affect CCSC niches [107].

#### 6.1.4. PI3K/AKT

The PI3K/AKT pathway is a central regulator of cell survival, metabolism, and proliferation. Activation of phosphoinositide 3-kinase (PI3K) leads to phosphorylation of AKT, which regulates multiple downstream targets involved in cellular growth, survival, and resistance to apoptosis [108].

In CRC, aberrant activation of the PI3K/AKT pathway commonly occurs through mutations in genes such as PIK3CA or through activation of upstream receptor tyrosine kinases, resulting in persistent pro-survival signaling [109]. Genetic and epigenetic alterations affecting PTEN, PIK3CA, and upstream HER family receptors have been shown to drive constitutive PI3K/AKT/mTOR activation in APC-driven models and human CRC, thereby cooperating with Wnt to sustain malignant growth [109,110]. This pathway contributes to CSC maintenance, promotes EMT, and enhances metastatic potential [30]. In CRC stem-cell-enriched cultures, inhibition of PI3K/AKT/mTOR signaling reduces sphere formation, induces apoptosis, and diminishes expression of stemness markers, indicating that this pathway is required for CCSC proliferation and survival [111].

Recent studies further demonstrate that PI3K/AKT signaling in CRC is tightly regulated at the post-transcriptional level by microRNAs (miRNAs) and non-coding RNAs, which modulate key components such as PTEN, PI3CA and AKT [112,113]. Multiple miRNAs have been associated with dysregulated expression of genes within the PI3K/AKT pathway in CRC, highlighting the role of epigenetic regulation in modulating pathway activity and tumor progression [114]. For example, several CSC-focused studies show that miRNA-mediated repression of negative regulators of PI3K/AKT enhances colonosphere formation, therapy resistance, and expression of CCSC markers such as CD44v6 [115,116].

Moreover, the PI3K/AKT signaling pathway does not function in isolation but interacts extensively with other oncogenic pathways. Crosstalk with Wnt/β-catenin, Hippo, MAPK/ERK, and TGF-β enables CRC cells to preserve proliferative and stem-like signaling even when individual pathways are pharmacologically inhibited, and PI3K-driven HER2/CD44v6 expression has been identified as a key axis sustaining CCSCs [117,118,119,120].

Thus, PI3K/AKT signaling contributes to colorectal tumorigenesis via integrated regulation by genetic mutations, epigenetic mechanisms, and signaling network interactions, supporting CSC survival and therapeutic resistance. Consistent with this, several groups have shown that pharmacologic or dietary inhibitors of PI3K/AKT/mTOR can decrease colorectal sphere-forming capacity, sensitize CCSC-enriched populations to chemo- and radiotherapy, and downregulate stemness-associated transcriptional programs [118,121].

#### 6.1.5. MAPK/ERK

The MAPK/ERK signaling pathway is another major regulator of cellular proliferation and differentiation. Activation typically occurs through upstream mutations or signaling events involving RAS and RAF proteins. This triggers a kinase cascade (RAF, MEK, ERK), that culminates in activation of extracellular signal-regulated kinase (ERK). Activated ERK translocates to the nucleus, where it regulates transcription factors controlling cell cycle progression and survival [122].

In CRC, mutations in oncogenes, including KRAS and BRAF, can lead to constitutive MAPK/ERK pathway activation, promoting uncontrolled cell proliferation and tumor progression. MAPK/ERK signaling also regulates key aspects of tumor aggressiveness, including cell survival, migration, and invasion [123]. Experimental work has shown that high MAPK activity defines a particularly aggressive subpopulation within heterogeneous CRC, and that ERK inhibition can reduce clonogenic growth and tumor-initiating capacity, implicating MAPK/ERK signaling in the maintenance of stem-like tumor cells [124]. Consistent with this, clinical data from first-line cetuximab plus FOLFIRI in metastatic CRC indicate that only tumors without activating KRAS or BRAF mutations derive a survival benefit, and that constitutive MAPK pathway activation can bypass EGFR blockade and is associated with poorer outcomes [125].

Studies have demonstrated that activation of MAPK/ERK signaling enhances the invasive and migratory capacity of CRC cells. In this regard, it has been shown that PBX3 promotes CRC cell migration and invasion through activation of the MAPK/ERK pathway, highlighting its role in metastatic progression [126]. More recently, intestinal-stem-cell-focused studies have shown that MAPK/ERK activity is essential for stem cell expansion and CRC initiation; for example, NSUN2-driven activation of ERK signaling promotes LGR5-positive intestinal stem cell expansion and APC-driven tumorigenesis, functionally linking MAPK/ERK to early CSC/CCSC-like compartments [124,127].

Furthermore, MAPK/ERK signaling has been directly linked to the regulation of cell proliferation and apoptosis in CRC. It has been demonstrated that activation of the MAPK/ERK pathway promotes proliferation while inhibiting apoptosis in colon cancer cells, further supporting its role in tumor growth and survival [128]. Within CSC-enriched models from other solid tumors, MAPK/ERK activity has been shown to support self-renewal and resistance to therapy [129,130], and similar mechanisms are proposed to operate in CCSCs [131].

Importantly, MAPK/ERK signaling interacts with other oncogenic pathways, including PI3K/AKT and Wnt signaling, to support CSC maintenance and tumor progression, reflecting the highly interconnected nature of signaling networks in CRC [132,133]. Crosstalk studies have demonstrated that Wnt/β-catenin activation can stabilize RAS and enhance MAPK signaling in CRC [134], while ERK and PI3K/AKT pathways converge on shared downstream effectors [135,136], providing multiple redundant routes to sustain CCSC survival and therapeutic resistance even when single pathways are inhibited.

#### 6.1.6. NF-κB

The NF-κB signaling pathway links inflammation to colorectal tumorigenesis and CSC maintenance [137]. NF-κB is a family of transcription factors that regulates genes involved in inflammation, immune responses, cell survival, and proliferation [138]. Aberrant activation of NF-κB signaling has been observed in colorectal tumors and is associated with increased tumor growth, resistance to apoptosis, and enhanced metastatic potential [137].

Studies in CSC-enriched models have shown that NF-κB activity supports self-renewal, clonogenic growth, and therapy resistance in stem-like tumor cell fractions, and that pharmacologic or genetic inhibition of NF-κB reduces CSC frequency and tumor-initiating capacity [139,140]. Building on this, Wang and colleagues demonstrated that the inflammatory mediator prostaglandin E2 drives CCSC expansion and liver metastasis in mice by activating NF-κB through EP4–PI3K and EP4–MAPK signaling, providing a mechanistic link between inflammation-induced NF-κB activation and CCSC maintenance in vivo [141]. Mesenchymal stem cells and other stromal components can likewise activate NF-κB in CRC cells through cytokine- and AMPK/mTOR-dependent signals, thereby enhancing sphere formation, epithelial–mesenchymal transition (EMT), and stem-like phenotypes in colon cancer cells [142]. Activation of NF-κB signaling also induces transcription of anti-apoptotic and pro-survival genes, including Bcl-2 family members, enabling tumor cells to evade cell death and sustain growth [143].

Beyond its role in CCSC maintenance, NF-κB signaling has been implicated in CRC progression and metastatic dissemination. Radiotherapy-induced activation of NF-κB is associated with poorer overall survival and increased distant metastasis in rectal cancer patients, while ROS-dependent NF-κB activation downstream of RAC1 promotes WNT-driven intestinal stem cell proliferation and CRC initiation, further supporting a role for NF-κB in sustaining aggressive stem-like tumor populations [144,145].

#### 6.1.7. TGF-β

The transforming growth factor-β (TGF-β) pathway plays a dual role in CRC. TGF-β can act as a tumor suppressor in early stages of disease but can also promote tumor progression, invasion, and metastasis in advanced cancers [146] TGF-β signaling is initiated by ligand binding to TGF-β receptors, leading to activation of Smad transcription factors that regulate genes involved in cell cycle arrest, differentiation, and apoptosis [147].

In early stages of tumorigenesis, TGF-β acts as a tumor suppressor by inhibiting epithelial cell proliferation and promoting apoptosis and its inactivation promotes colorectal tumorigenesis [146]. However, as CRC progresses, tumor cells often develop resistance to these growth-inhibitory effects, allowing TGF-β signaling to shift towards a pro-tumorigenic role [146,148].

In advanced CRC, TGF-β signaling promotes tumor progression through effects on both tumor cells and the surrounding microenvironment [147,149]. It has been demonstrated that CRC depends on a TGF-β- driven program in stromal cells to initiate metastasis, highlighting the importance of paracrine signaling between tumor and stromal compartments. The stromal activation enhances tumor invasion and dissemination, emphasizing that TGF-β signaling is a key regulator of metastatic progression [150].

More recent studies have shown that TGF-β signaling contributes to the maintenance of CSC properties by promoting cellular plasticity and enabling tumor cells to acquire stem-like phenotypes [40,147]. For example, Nakano et al. demonstrated that TGF-β signaling is specifically activated in undifferentiated CD44-positive CRC cells and that exogenous TGF-β, via TWIST1 induction, can convert CD44-negative non-CSCs into CD44-positive CSCs, thereby increasing the CSC pool in xenograft models [151]. Complementary mechanistic work has identified a positive ALG10/TGF-β feedback loop in which TGF-β–dependent glycosylation of TGFBR2 sustains pathway activity and is required for CRC stemness, directly linking TGF-β signaling to CCSC maintenance [152]. Collectively, these findings suggest that TGF-β signaling expands the CSC pool, enhances tumor heterogeneity, and promotes metastatic potential and therapeutic resistance. Consistent with broader EMT–stemness models, many of these effects are mediated through TGF-β–induced EMT and epithelial–mesenchymal plasticity, which endow carcinoma cells with CSC-like traits [153]. Non-coding RNAs, alternative splicing, and epigenetic mechanisms further fine-tune this TGF-β-driven CSC program across tumor types [154,155].

#### 6.1.8. JAK/STAT3

The JAK/STAT3 signaling axis is a central mediator of inflammatory and cytokine-driven responses in CRC and has emerged as a key regulator of CCSC maintenance, tumor–microenvironment interactions, and therapeutic resistance. In this pathway, engagement of cytokine receptors (such as those for IL-6, IL-11, and other gp130-associated ligands) activates Janus kinases (JAKs), which phosphorylate STAT3, enabling its dimerization and nuclear translocation to drive transcription of genes involved in stemness, survival, proliferation, and immune modulation [156]. In normal intestinal tissue, transient STAT3 activation contributes to epithelial regeneration after injury, whereas in CRC, persistent JAK/STAT3 signaling is frequently observed in CCSC-enriched populations and correlates with poor prognosis and chemoresistance [157,158].

Multiple studies have shown that IL-6–driven STAT3 activation promotes CCSC phenotypes in CRC models, increasing sphere formation, expression of stemness markers (such as CD44, CD133, and LGR5), and resistance to fluoropyrimidines and oxaliplatin [28,159,160]. TAMs, CAFs, and mesenchymal stromal cells secrete IL-6 and related cytokines within the TME, creating a paracrine loop in which stromal JAK/STAT3 activation sustains pro-tumorigenic cytokine production while tumor-cell STAT3 drives CCSC maintenance and immune evasion [161,162]. This bidirectional crosstalk links inflammatory signals directly to CCSC expansion and illustrates how JAK/STAT3 integrates intrinsic stemness programs with extrinsic niche factors.

JAK/STAT3 signaling also contributes to EMT, angiogenesis, and metastatic spread, further embedding CCSC biology within broader malignant processes [163]. STAT3 target genes include key regulators of anti-apoptotic and pro-survival pathways (such as BCL-XL and survivin), as well as transcriptional programs associated with invasion and dissemination, providing a mechanistic basis for the role of JAK/STAT3 in therapy resistance [164]. Consistent with this, pharmacologic inhibition of JAK or STAT3 in CCSC-enriched cultures reduces sphere formation, downregulates stemness-associated transcripts (including NANOG and SOX2), and restores sensitivity to chemotherapy and radiotherapy, supporting JAK/STAT3 as a tractable CCSC vulnerability [158,165].

Therapeutically, JAK/STAT3 targeting has been explored using small-molecule inhibitors and agents such as napabucasin, which suppresses STAT3-driven transcription and reduces expression of stemness-associated genes [166]. Preclinical data indicate that STAT3 inhibition can impair stem-like tumor cell populations and potentiate chemotherapy in gastrointestinal (GI) models, prompting multiple clinical evaluations in metastatic CRC [167].

#### 6.1.9. Signaling Crosstalk in CCSC

Rather than functioning in isolation, key signaling pathways governing CCSC biology exhibit extensive molecular crosstalk that coordinates stemness, cellular plasticity, survival, proliferation, EMT, and inflammatory responses. These reciprocal interactions establish a robust signaling network that enables CCSCs to adapt to microenvironmental and therapeutic stress while driving tumor progression.

**Figure 3:**
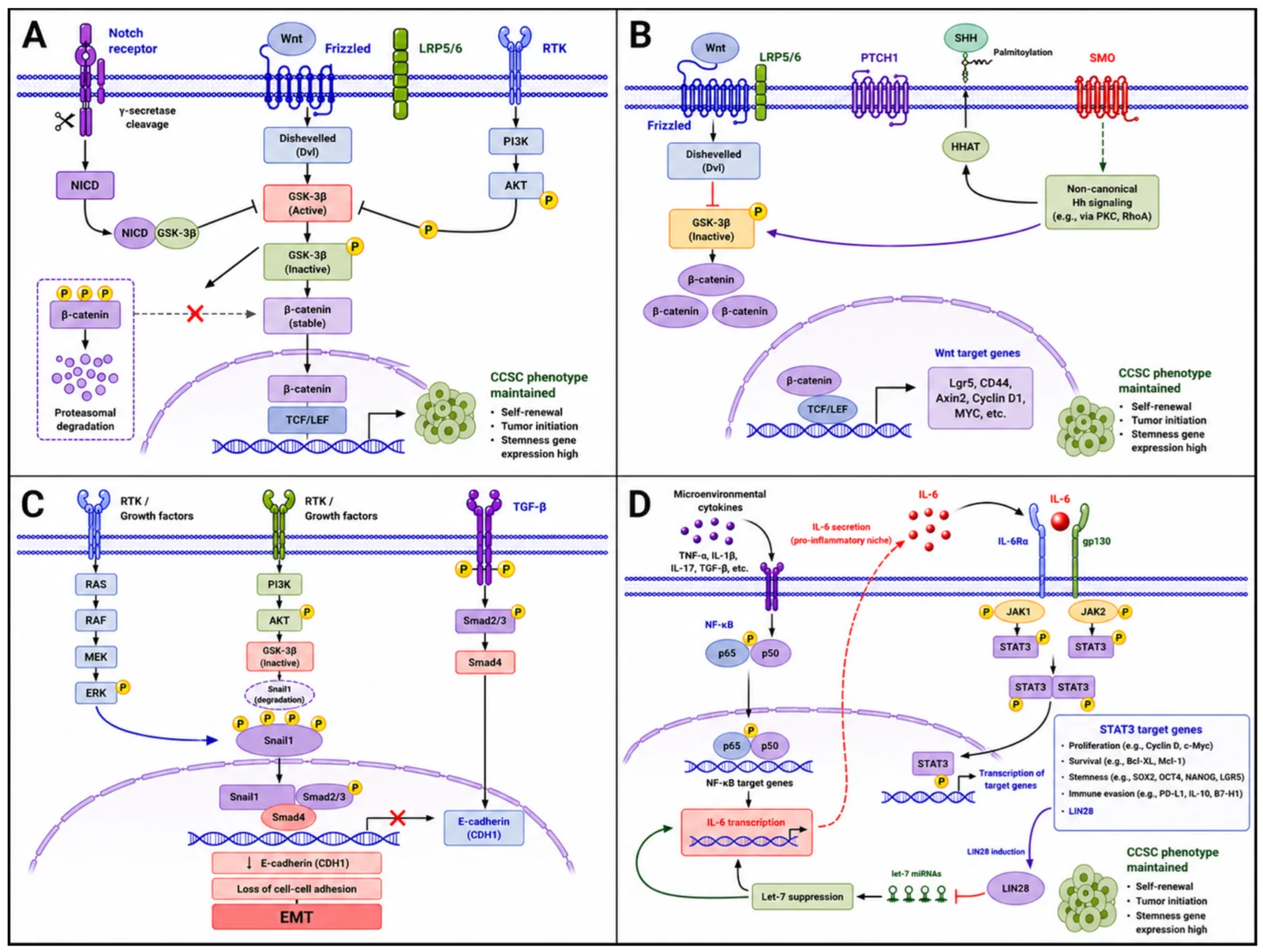
Crosstalk Among Oncogenic Signaling Pathways in CCSCs: (**A**) Notch–Wnt–PI3K/AKT crosstalk regulating β-catenin stabilization. (**B**) Wnt–Non-canonical Hedgehog crosstalk sustaining Wnt/β-catenin signaling. (**C**) MAPK/ERK–PI3K/AKT–TGF-β crosstalk promoting EMT. (**D**) NF-κB–IL-6/JAK/STAT3 crosstalk sustaining inflammatory stemness signaling. Shenoy, A. (2026) https://BioRender.com/p14e8lv.

Mechanistically, these signaling pathways cooperate through multiple nodes of convergence that collectively reinforce the CCSC phenotype (Figure 3). For instance, Notch-released NICD binds and sequesters GSK-3β, while hyperactivated AKT (from the PI3K/AKT pathway) directly phosphorylates and inactivates it; both actions prevent β-catenin degradation, allowing it to freely accumulate and enter the nucleus independent of Wnt ligands [100,117,168]. Regan et al. demonstrated that non-canonical Hh signaling maintains Wnt/β-catenin activity in CCSCs. Pharmacological inhibition of HHAT (the enzyme required for SHH palmitoylation and ligand activation) or suppression of SHH impaired Hh signaling, resulting in reduced Wnt signaling, decreased β-catenin-dependent transcription, diminished expression of Wnt target genes, and subsequent loss of the CSC phenotype [106]. Concurrently, the MAPK/ERK and PI3K/AKT pathways converge to stabilize Snail1 through complementary post-translational mechanisms. PI3K/AKT stabilizes Snail1 by inhibiting GSK-3β-mediated degradation, while ERK-mediated phosphorylation enhances Snail1 stability, nuclear retention, and transcriptional activity [117,133]. The resulting accumulation of nuclear Snail1 enables the formation of a transcriptional repressor complex with TGF-β-activated Smad3/4 to suppress E-cadherin expression, while TGF-β-induced GLI2 signaling further reinforces EMT through complementary transcriptional programs. Together, these interconnected pathways drive EMT required for CCSC migration and distant metastasis [76,101,147,169]. Simultaneously, a potent microenvironmental feedback loop is driven by the convergence of developmental and inflammatory pathways, rendering CCSCs highly adaptable and resistant to single-agent therapies. Microenvironmental cytokines activate NF-κB, which directly drives the secretion of Interleukin-6 (IL-6), subsequently triggering the JAK/STAT3 pathway [141,156,160]. Phosphorylated STAT3 then upregulates LIN28, which suppresses the let-7 microRNA, a negative regulator of IL-6, locking the cells into a continuous, self-sustaining loop of chronic inflammation and stemness [160,170]. Furthermore, TGF-β/Smad3 signaling completely bypasses canonical Hh receptor constraints by directly binding to the GLI2 promoter, ensuring that Gli-mediated survival genes remain active even during treatment with SMO inhibitors [76,171]. Together, the convergence of STAT3, NF-κB, Smads, and Gli proteins on the promoters of pluripotency genes like NANOG, SOX2, and OCT4, alongside anti-apoptotic factors like BCL2, creates a redundant survival matrix [76,133,160,169,170,171]. This collective network ensures that if one pathway is pharmacologically blocked, alternative inputs immediately compensate to sustain the CCSC phenotype.

### 6.2. Epigenetic Regulation and Transcriptional Control

CCSC behavior is also regulated by epigenetic mechanisms and transcriptional programs that control gene expression without altering the underlying DNA sequence. Epigenetic modifications such as DNA methylation, histone modifications, and chromatin remodeling play critical roles in maintaining stemness and regulating differentiation in CRC cells [172,173]. A 2023 study reported that CCSC-enriched spheres and xenografts expressed higher levels of DNMT1 and showed broader promoter hypermethylation than bulk tumor cells, and that DNMT1 inhibition with 5-Aza-dC reduced CSC abundance and prevented liver metastatic growth in mouse models [174].

Key transcription factors associated with stemness include SOX2, OCT4, NANOG, and MYC. The expression of these transcription factors is strongly regulated via epigenetic regulators, which also influence major signaling pathways such as Wnt and Notch, thereby reinforcing stem-cell-like properties in tumor cells [175]. Chen et al. showed that EZH2-mediated H3K27 trimethylation drives CRC stem-like cell expansion by repressing p21^CIP1^, thereby facilitating cell-cycle progression and activating Wnt/β-catenin signaling, while genetic or pharmacologic EZH2 inhibition reduces sphere formation and tumorigenicity [176]. Building on this, Ghobashi et al. demonstrated that PI3K/AKT-dependent phosphorylation of EZH2 enhances EZH2-driven β-catenin trimethylation, further hyperactivating Wnt target genes and reinforcing tumor stemness in CRC [177]. Recent work also shows that loss of the intestinal transcription factors CDX1 and CDX2 increases expression of LGR5 and CD44 and enhances colon cancer stemness, in part by altering β-catenin–dependent transcriptional complexes at stemness-associated loci, highlighting how lineage transcription factors and chromatin context shape CCSC programs [178].

Importantly, epigenetic regulation contributes to cellular plasticity, allowing non-cancer stem cells to revert to a stem-like state under specific conditions [179]. Studies have demonstrated that microenvironment signals can modulate epigenetic states and activate stemness-associated transcription programs, highlighting the dynamic interplay between signaling pathways and epigenetic control mechanisms [179]. For instance, TGF-β-driven dedifferentiation of human CRC cells enhances CD44-positive CSC properties through EMT-associated transcriptional and epigenetic reprogramming, while DNA methyltransferase inhibition restores RARB expression, reduces CSC traits, and sensitizes radio-resistant CRC cells to radiotherapy [174]. MicroRNA-based work has defined a clear, post-transcriptional layer of CCSC control centered on miR-221. Mukohyama et al. showed that miR-221 is overexpressed in CCSCs, directly targets and suppresses the RNA-binding protein QKI, and thereby enhances organoid-forming capacity and tumorigenicity [50]. Building on this, another study reported that constitutive miR-221 knockdown markedly reduces stemness and tumor formation in patient-derived xenograft models [180]. A recent study further demonstrated that therapeutic inhibition of miR-221 with locked nucleic acid (LNA) antisense oligonucleotides impairs colorectal tumor growth in vivo, establishing miR-221 as a key epigenetic regulator and actionable vulnerability in CCSCs.

This epigenetic plasticity reinforces the microenvironment-driven CCSC state transitions described in prior sections, thereby sustaining heterogeneity and therapy resistance [179]. These findings highlight the complexity of CCSC regulation and identify epigenetic mechanisms as promising targets for therapeutic intervention aimed at eliminating CSC populations [181]. Accordingly, epigenetic-based strategies, such as DNMT and HDAC inhibitors, or therapies directed against CSC-regulating miRNAs and lncRNAs, are being explored to deplete CCSCs or resensitize them to conventional treatments in CRC [182,183,184].

At the same time, epigenetic and transcriptional regulators integrate inputs from Wnt/β-catenin, Notch, PI3K/AKT, MAPK/ERK, NF-κB, and TGF-β signaling, enabling CCSCs to dynamically adapt to environmental and therapeutic pressures and contributing to pathway-level redundancy that complicates targeted intervention [173,179,185].

## 7. Diagnostic and Prognostic Implications

CCSCs have emerged as important contributors to the diagnosis, prognosis, and clinical management of CRC [186,187]. Because CCSCs are associated with tumor initiation, metastasis, and therapy resistance, biomarkers linked to these cells may provide clinically useful information regarding disease aggressiveness and patient outcomes [30,188].

A variety of surface markers and stemness-associated proteins have been investigated for their diagnostic and prognostic significance in CRC, including CD133, CD44, LGR5, ALDH1, EpCAM, and BMI1 [29,53,189,190] (Table 1). Elevated expression of these markers has frequently been associated with advanced tumor stage, lymph node involvement, metastatic disease, and reduced overall survival [28,191]. However, comparative studies indicate that not all markers carry equivalent prognostic weight; for example, LGR5 and ALDH1 frequently demonstrate a significantly stronger correlation with aggressive clinicopathological behavior than CD44 or EpCAM [29,52,192], whereas loss of multiple adhesion-related markers (CD44, CD166, EpCAM) can itself mark progression [49]. Marker expression can vary across tumor subtypes and may reflect the dynamic plasticity of CCSC populations [193].

Among these markers, LGR5 has gained particular attention as both a marker of normal intestinal stem cells and a clinically relevant indicator of aggressive tumor behavior. Increased LGR5 expression has been associated with enhanced tumor growth, recurrence risk, and metastatic potential in several CRC cohorts [20,34]. Meta-analytic data further support that LGR5 overexpression is significantly associated with reduced overall survival, reinforcing its value as a stemness-linked prognostic biomarker [194]. Similarly, elevated CD44 and CD133 expression have been correlated with poor differentiation, increased invasiveness, and unfavorable prognosis [195,196]. Consistent with this observation and complexity outlined in Section 3.3, loss of CD44v6 expression has also been reported as an independent predictor of poor clinical outcome in specific cohorts, highlighting that the prognostic impact of CD44-associated signaling in CRC tumor progression may depend on biological context and analytical methodology [38].

Beyond single markers, gene expression signatures and molecular profiling approaches that capture stemness-related programs may offer improved prognostic value [197]. Tumors enriched for CSC-associated transcriptional profiles often demonstrate increased metastatic potential, resistance to therapy, and shorter survival. Recent work integrating CSC-related signatures with consensus molecular subtypes (CMS) and other genomic classifiers suggests that stemness-high CRCs cluster in poor-prognosis groups and may benefit from intensified or CSC-directed treatment strategies [197,198]. Integration of CCSC biomarkers with established clinicopathologic factors and molecular classifications may therefore improve risk stratification and treatment selection.

CCSC-associated markers may also have diagnostic utility through liquid biopsy approaches. Detection of circulating tumor cells, extracellular vesicles, or cell-free nucleic acids expressing stemness-associated markers could facilitate earlier detection of recurrence or monitoring of treatment response [199,200]. In this context, tumor-derived EVs are being evaluated as liquid biopsy biomarkers, because their cargo can report on CSC burden, evolving resistance pathways, and pre-metastatic niche activity in a minimally invasive manner [201]. For instance, peripheral-blood detection of CEA/CK/CD133 mRNA—reflecting circulating stem-like tumor cells—identifies Dukes’ B and C CRC patients at significantly higher risk of recurrence and poor overall and disease-free survival, supporting the use of CCSC-like CTC signatures as non-invasive prognostic tools [196,199,200]. Although these strategies remain under active investigation, they represent promising noninvasive tools for precision oncology.

Despite their potential, several challenges remain before CCSC biomarkers can be routinely implemented in clinical practice. Marker heterogeneity, intratumoral plasticity, and lack of assay standardization currently limit reproducibility across stud. Nevertheless, continued refinement of CCSC-based diagnostic and prognostic models may improve personalized management of CRC patients.

## 8. CCSC-Mediated Therapy Resistance

CCSCs are widely implicated in resistance to chemotherapy, radiotherapy, and targeted therapies, contributing to tumor persistence, recurrence, and poor clinical outcomes. Unlike the bulk tumor population, CCSCs possess stem-like properties that enable survival under therapeutic stress, allowing them to regenerate tumors following treatment [202,203]. One major mechanism of therapy resistance is the relatively quiescent or slow-cycling state of CCSCs. Because many cytotoxic therapies preferentially target rapidly proliferating cells, quiescent CCSCs may evade treatment and remain viable after therapy [203,204].

CCSCs also exhibit enhanced expression of ATP-binding cassette (ABC) drug transporters, including ABCB1 and ABCG2, which actively efflux chemotherapeutic agents and reduce intracellular drug accumulation. Increased transporter activity has been associated with resistance to agents commonly used in colorectal cancer treatment, such as 5-fluorouracil and oxaliplatin [131,205]. In addition, CD133-positive CCSCs can secrete IL-4 in an autocrine fashion to protect themselves from chemotherapy-induced apoptosis, and neutralization of IL-4 has been shown to resensitize these cells to cytotoxic agents in vitro and in vivo [6]. Moreover, CCSCs possess robust DNA damage repair mechanisms and resistance to apoptosis, enabling survival after exposure to chemotherapy or radiation. Activation of pathways such as PI3K/AKT, NF-κB, and Wnt/β-catenin promotes anti-apoptotic signaling and cellular recovery following treatment-induced stress [131].

As outlined in the TME section, stromal cells, inflammatory cytokines, hypoxia, and extracellular matrix interactions activate Notch, Hedgehog, TGF-β, and other pathways that reinforce CCSC-mediated therapy resistance [206].

Importantly, CCSCs display substantial cellular plasticity, allowing non-stem cancer cells to dedifferentiate into stem-like cells after treatment. This dynamic interconversion contributes to residual disease and may explain why initial therapeutic responses are often followed by relapse [207]. Together, these intrinsic and microenvironment-driven mechanisms underscore the need for therapeutic strategies that explicitly target CSC populations rather than focusing solely on the bulk tumor compartment.

## 9. Therapeutic Targeting of CCSCs

CCSC-directed strategies build on prior evidence that these cells survive conventional therapy and regenerate tumors, motivating approaches that target CCSC survival, self-renewal, and plasticity. Inhibiting Stemness-associated signaling pathways discussed above can disrupt the core programs that sustain CCSC populations [208]. Preclinical studies show that targeting these pathways reduces colonosphere formation, depletes LGR5^+^/CD44^+^ CSC-like fractions, and sensitizes CCSCs to cytotoxic therapies [209]. Experimental work also indicates that plasticity-driven state transitions are at least partly reversible. Stem-like cells can differentiate when key pathways such as Wnt, Notch, TGF-β, or Hippo–YAP are attenuated [18]. Early preclinical studies suggest that targeting CCSC-associated metabolic reprogramming (including glycolysis, mitochondrial oxidative metabolism, and lipid pathways) may complement pathway-directed therapies by weakening stem-like tumor cells and enhancing treatment responses [81]. Therapy-induced stem-cell and regenerative programs in CRC, including revival and fetal-like states, are driven by cellular plasticity and microenvironmental signals such as TGF-β and YAP, and may be attenuated if these regenerative cues are reduced or pharmacologically disrupted [210,211,212]. Together, these observations suggest that CCSC plasticity often reflects an epigenetic and niche-dependent state rather than an irreversible genetic change. Coping with this behavior therefore requires therapeutic strategies that both deplete pre-existing CCSCs and limit reprogramming of non-stem cells. For example, cytotoxic or anti-EGFR regimens can be combined with inhibitors of plasticity-regulating nodes (such as MET–STAT3, YAP, or AURKA) [213,214] and with interventions that modulate hypoxia and stromal cytokine networks to reduce the microenvironmental signals that drive dedifferentiation.

Beyond targeting signaling pathways and niche-dependent plasticity, additional therapeutic strategies are emerging to disrupt the molecular mechanisms that sustain CCSCs. Given the dynamic interactions between CCSCs and their microenvironment, durable therapeutic responses will likely require combination approaches that not only eliminate existing CCSCs but also disrupt the niche-dependent signals that drive dedifferentiation and stem-cell reconstitution [215]. Therapeutic targeting of epigenetic regulators has also gained attention. Because DNA methylation, histone modification, chromatin remodeling, and noncoding RNAs contribute to CCSC maintenance, epigenetic therapies may disrupt stemness programs and restore treatment sensitivity [216]. For instance, inhibiting DNMT1 or EZH2 reduces CCSC self-renewal and metastatic capacity in vivo [217], while miRNA-directed strategies (such as anti-miR-221) diminish CSC frequency and tumor growth, indicating the importance of epigenetic and post-transcriptional regulation as actionable CCSC vulnerabilities [50]. In parallel, recent studies have highlighted the potential value of targeting transcriptional regulators and stemness-associated effectors, including newer candidate targets such as SMYD3, which has been proposed as a direct regulator of CRC stem-cell proliferation through effects on c-MYC-related programs [218].

Another promising area is CCSC-directed immunotherapy. Preclinical work with LGR5-targeted antibody–drug conjugates, and antibodies against CSC-associated antigens such as CD44 variants, shows that selectively attacking CCSC populations can eradicate tumors and delay recurrence in xenograft models [219]. In GI and colon cancer models, anti-LGR5 antibody–drug conjugates (ADC) have been shown to shrink LGR5^high^ tumors, eradicate CSC-rich lesions, and prevent relapse, providing direct proof-of-concept for CSC-targeting ADCs [220]. CD44, particularly CD44v isoforms, has similarly been validated as a functional CSC marker and therapeutic target across solid tumors, with antibody-based interference reducing stem-like traits and invasive behavior [221]. Beyond antibody-based approaches, emerging CCSC-relevant immunotherapies include immune checkpoint blockade, which targets inhibitory molecules upregulated on CCSC-like cells or within CCSC-rich niches to restore T-cell activity [222]. CAR-T and CAR-NK constructs engineered against CRC/CCSC antigens such as EpCAM, CD166, and PD-L1 have shown the ability to directly lyse stem-like tumor cells in preclinical models [223,224,225]. In addition, macrophage-reprogramming and dendritic-cell (DC)/CSC vaccine strategies, such as CCSC-derived antigen-loaded DC vaccines, have demonstrated synergistic tumor control when combined with PD-L1-CAR-T cells in CRC models [225].

Several therapeutic approaches targeting CSC-associated pathways are currently under preclinical and clinical investigation. Porcupine inhibitors, which block Wnt ligand secretion, and small-molecule inhibitors of the Wnt/β-catenin interaction have entered early-phase clinical trials in patients with advanced solid tumors, including CRC. However, translating these approaches into effective clinical therapies has proven challenging; for example, Zhong and Virshup demonstrated that recurrent mutations in the tumor suppressor FBXW7, found in approximately 15% of CRCs, enable cancer cells to bypass Wnt/β-catenin dependence entirely, rendering Wnt pathway inhibitors ineffective in this patient subset [226]. Additional challenges include tumor heterogeneity, CSC plasticity, compensatory signaling mechanisms, and the substantial overlap between signaling pathways that regulate CCSCs and those that maintain normal intestinal stem cells. Pathways such as Wnt/β-catenin, Notch, Hedgehog, PI3K/AKT/mTOR, and TGF-β/BMP all contribute to both crypt stem-cell homeostasis and CCSC maintenance, so broad pathway inhibition carries a risk of damaging the normal stem-cell compartment [79]. This issue is particularly critical for LGR5-directed strategies and Wnt pathway inhibition, because LGR5 marks both malignant CCSCs and homeostatic crypt stem cells, and canonical Wnt/β-catenin signaling is essential for normal crypt maintenance. As a result, CCSC-targeted interventions that deplete LGR5^+^ cells or strongly suppress Wnt signaling risk dose-limiting intestinal toxicity [20,227]. Translational development of CCSC-directed therapies will therefore require approaches that either exploit differential pathway dependencies, use highly selective delivery platforms (such as tumor-restricted CAR-T, bispecifics, or ADCs), or incorporate safety mechanisms that spare normal stem cells while effectively targeting malignant CCSC populations.

## 10. Clinical Trials

A structured search of ClinicalTrials.gov was performed (database cutoff: 30 July 2026; records verified against the last-update dates shown in Table 2) to identify interventional studies related to CCSC biology and stemness-associated signaling in colorectal cancer. The search query combined colorectal cancer terms (“colorectal cancer,” “colon cancer,” or “rectal cancer”) with CCSC-related terms (“cancer stem cell,” “stemness,” “stem cell marker,” CD133, CD44, ALDH, or LGR5) and was restricted to interventional studies. No additional eligibility filters were applied. Studies were included if the registered record explicitly referenced a CSC marker, a stemness-associated pathway, or a CCSC-directed therapeutic or biomarker objective, and were excluded only when CSC-related terminology appeared in an unrelated context. All 19 eligible studies were included and categorized according to their primary translational focus: (i) CCSC marker-targeted therapies, (ii) stemness pathway-targeted therapies, (iii) biomarker/window-of-opportunity studies, (iv) prevention studies, and (v) other CCSC-related translational studies. Trials with overlapping features were classified according to their primary registered endpoint.

Among the most direct CSC-targeted approaches, a completed phase 1/2 trial (NCT02176746) evaluated an autologous CSC vaccine targeting ALDH^+^ CSC-restricted antigens in patients with metastatic colorectal adenocarcinoma, using adverse events as the primary endpoint. This study represents one of the earliest attempts to exploit CSC-restricted antigenicity for active immunotherapy in CRC. It demonstrates that CSC-directed vaccination is feasible and generally tolerable but also illustrates how challenging it is to achieve durable, clinically meaningful anti-CSC responses in heavily pretreated metastatic disease, particularly as no efficacy data reported a decade after completion. A separate phase 1 trial (NCT01440127) evaluated short-course metformin pretreatment in resectable CRC, with CD133 expression in tumor tissue as a pharmacodynamic readout of CCSC burden. Although terminated due to poor accrual, the pre-surgical safety experience was subsequently reported [235]. The study illustrated the rationale for repurposing metabolic agents, as metformin has been shown to inhibit mitochondrial complex I, activate AMPK, and reduce CD133+ TIC populations in preclinical CRC models [236]. It also emphasized practical barriers such as recruitment and the complexity of interpreting CD133 as a sole surrogate for stem-cell burden in the clinic. A further study frames the limits of CD133 as a target. CART133 (NCT02541370) established that CD133^+^ cells can be engaged by engineered T cells, with efficacy and biomarker data reported for the hepatocellular carcinoma cohort [229]. However, no colorectal-specific results have been published, and no outcomes are posted on ClinicalTrials.gov, so CRC inference remains limited. More broadly, CD133 is also expressed by normal regenerative progenitors, so marker-directed strategies must contend with shared stem-cell biology in non-malignant tissue.

More recent trials reflect a shift toward marker-directed cellular and antibody-based therapies. A recruiting phase 1/phase 2a trial (NCT05759728) is evaluating CNA3103, an autologous CAR-T cell therapy directed against LGR5, in adults with metastatic CRC. LGR5 is a Wnt target gene and intestinal stem cell marker that potentiates Wnt/β-catenin signaling through R-spondin binding, and its expression is required at screening for enrollment. The primary endpoints include safety and best overall response. Therefore, early readouts will primarily inform the tolerability of directly targeting an intestinal stem cell-associated antigen and the feasibility of enrolling biomarker-selected patients, rather than definitive efficacy. A separate recruiting phase 1/phase 2 trial (NCT03526835) is evaluating MCLA-158 (petosemtamab), a bispecific antibody targeting EGFR and LGR5 that directs EGFR degradation specifically on LGR5+ CSCs, as monotherapy or in combination regimens in advanced solid tumors including metastatic CRC [228]. This study will help clarify whether dual targeting of a lineage driver (EGFR) and a stemness marker (LGR5) can translate organoid and xenograft activity into clinical benefit and will also probe the balance between on-target CSC effects and potential off-tumor toxicity. A third LGR5-directed agent, the monoclonal antibody BNC101 (NCT02726334), completed monotherapy dose escalation but was terminated before the FOLFIRI combination arm proceeded, indicating that attrition in this space reflects sponsor and portfolio decisions as much as biology. Together, these trials position LGR5 as the most clinically actionable CCSC-associated marker in current CRC development.

Stemness-pathway-directed therapeutics have also been evaluated in larger randomized settings. Napabucasin, an orally administered inhibitor of STAT3-driven transcription that reduces expression of stemness-associated genes including NANOG and β-catenin targets, was tested in two phase 3 trials in metastatic CRC (NCT02753127 and NCT03522649) in combination with FOLFIRI, with overall survival as the primary endpoint [230]. In CanStem303C (NCT02753127), the addition of napabucasin to FOLFIRI did not improve overall survival compared with FOLFIRI alone [230], despite robust preclinical data and earlier signals in biomarker-enriched cohorts, illustrating how pathway redundancy, tumor-cell plasticity, and incomplete biomarker selection can blunt the clinical impact of CSC-targeted stemness inhibitors [230]. The parallel phase 3 study (NCT03522649) has not been reported, so its contribution remains uncertain. Notch/DLL4 blockade forms a second pathway-directed strand: demcizumab with FOLFIRI (NCT01189942) completed dose finding and the anti-DLL4/VEGF bispecific OMP-305B83 (NCT03035253) was terminated, neither with a published report, consistent with the narrow therapeutic index of sustained Notch inhibition. Mithramycin (NCT02859415) was likewise terminated for insufficient accrual. Conversely, ONC201/dordaviprone (NCT05630794) has entered phase 1 evaluation in adenomatous polyps, familial adenomatous polyposis, and CRC, interrogating CD133, CD44, ALDH, and LGR5, and extending pathway-directed strategies into the precancerous setting. Additional pathway-oriented studies included a pre-operative window trial of decitabine in colon cancer (NCT01882660), which measured Wnt target gene expression (APCDD1, AXIN2, DKK1, LGR5, ASCL2) as a primary pharmacodynamic endpoint. In the small terminated cohort, the study showed no consistent demethylation or induction of Wnt target genes in resected tumors [231]. Another study evaluated mesalazine in a chemoprevention setting (NCT02077777) to investigate its effects on β-catenin signaling in colorectal mucosa. These smaller studies demonstrate that stemness and Wnt-related readouts can be incorporated into trial design as pharmacodynamic endpoints, but they also show that modulation of pathway signatures does not automatically translate into clear clinical benefit and that larger, prospectively validated biomarker strategies will be required.

A further group used CSC markers as readouts rather than targets. A CD133-based chemosensitivity assay (NCT01483001) proved technically feasible in patients with lung cancer and CRC but did not establish predictive validity [232]. Similarly, NCT03085992 applied CD133 as a correlative marker alongside a disease-free survival endpoint in resectable rectal cancer [233]. In contrast, NCT00960427, which was designed to correlate post-chemoradiotherapy changes in stem cell markers with disease-free survival, was withdrawn before enrolling because no surgeon was available for the required sampling. Collectively, these studies show that CSC-resolved correlative endpoints are attractive but operationally fragile.

Prevention-oriented trials further extend CCSC biology into the early-disease setting. The ASPIRED-XT trial (NCT05056896) is evaluating low-dose aspirin as a chemopreventive agent, with change in intestinal stem cell marker gene expression, specifically LGR5+ intestinal stem cells, as a primary outcome measured by single-cell transcriptomics. It extends the parent ASPIRED trial, which demonstrated dose-dependent suppression of PGE_2_ biosynthesis in individuals with colorectal adenomas [234]. This represents one of the few CRC trials to apply stem cell-resolved molecular endpoints in a chemoprevention context. Its results will be informative for understanding whether modulation of intestinal stem cell programs is a realistic, measurable surrogate for long-term risk reduction in high-risk populations.

Collectively, these trials reveal persistent translational barriers, particularly slow accrual in molecularly selected populations and logistical constraints, which are examined in greater detail in the subsequent section on translational barriers.

## 11. Emerging Technologies and Future Directions

Building on the CCSC-directed clinical trial landscape described above, several emerging technologies are positioned to advance stem cell-targeted translation in CRC beyond the limitations of current approaches. In particular, precision oncology-guided combination therapies, patient-derived organoid platforms, and genome-scale CRISPR screens offer complementary routes to refine CCSC-directed interventions and select patients most likely to benefit.

### 11.1. Next-Generation LGR5-Directed Platforms

LGR5-directed CAR-T and bispecific antibody strategies are the most clinically advanced CCSC-targeting platforms in current CRC development, exemplified by CNA3103 (NCT05759728) and MCLA-158 (NCT03526835). Their continued development will depend on overcoming on-target, off-tumor toxicity against normal intestinal stem cells, which also express LGR5 as part of physiological crypt regeneration. Emerging cellular-engineering strategies, including logic-gated dual-antigen constructs, inducible safety switches, and regional delivery, may improve tumor selectivity, controllability, or local exposure. CRC-specific preclinical work has demonstrated AND-gated CEA/EpCAM adaptor CAR-T-cell activity [237], while inducible caspase-9 safety switches can enable pharmacologic elimination of infused T cells during severe toxicity [238], and intraperitoneal CAR-T administration has shown enhanced activity in preclinical models of CRC peritoneal carcinomatosis. In a precision oncology framework, such platforms will likely be deployed in biomarker-selected subgroups (for example, LGR5^high^, CEA/EpCAM-coexpressing tumors), guided by integrated tissue and circulating DNA (ctDNA) profiling [239]. For bispecific antibodies such as MCLA-158, longitudinal tissue and ctDNA profiling may help identify emerging resistance and tumor heterogeneity during treatment. Although not yet validated specifically for MCLA-158, serial ctDNA and paired tissue–plasma analyses in EGFR-targeted CRC therapy have detected evolving resistance alterations and informed anti-EGFR treatment decisions [240,241].

### 11.2. Single-Cell and Pharmacodynamic Biomarker Strategies

The ASPIRED-XT trial (NCT05056896) illustrates a shift toward stem cell-resolved molecular endpoints, applying single-cell transcriptomic measurement of LGR5+ intestinal stem cell populations as a primary outcome. Single-cell approaches enable direct quantification of stem-like transcriptional programs and can distinguish malignant stem-like cells from normal intestinal stem cells, capabilities that conventional bulk assays cannot provide. Complementary pharmacodynamic studies, including the metformin/CD133 trial (NCT01440127) and the decitabine/Wnt target gene trial (NCT01882660), demonstrate the feasibility of integrating CCSC marker readouts into window-of-opportunity designs while leveraging repurposed agents with established safety profiles. More broadly, embedding CCSC-resolved single-cell and spatial profiling into precision oncology pipelines may enable real-time tracking of CCSC abundance, state transitions, and microenvironmental interactions during combination therapy. Broader adoption of these approaches will require standardized, clinically validated assays for CD44, CD133, ALDH, and LGR5 supported by centralized laboratory infrastructure.

### 11.3. Personalized Vaccines and Immunotherapy Combinations

Building on the autologous CSC vaccine concept (NCT02176746), future development is extending toward personalized neoantigen platforms delivered as peptides, dendritic-cell vaccines, or mRNA-based products [242]. A preliminary clinical study has demonstrated the feasibility of personalized neoantigen vaccination in microsatellite-stable (MSS) advanced CRC [243]. The principal translational challenge lies in achieving meaningful immune activation within the immunosuppressive microenvironment of microsatellite-stable CRC, which will likely require integration with checkpoint blockade or microenvironment-modulating combinations [244,245]. Rational combination designs that pair CCSC-directed vaccines or CAR-T/CAR-NK constructs with agents that remodel the TME (for example, myeloid-targeting or stromal-modulating therapies) may prove particularly important in CCSC-rich, immune-excluded tumors [246,247].

### 11.4. Distinguishing Malignant Stemness from Normal Intestinal Biology

A recurring biological challenge across all emerging platforms is the overlap between malignant stem-like programs and physiological intestinal stem cell biology. LGR5, the most clinically actionable CCSC marker to date, is also essential for normal crypt regeneration, and many of the signaling pathways that sustain CCSC self-renewal are simultaneously required for intestinal homeostasis [248,249]. Future clinical translation will require correlative endpoints that can distinguish true disruption of malignant stem-like populations from damage to normal regenerative compartments. This distinction is unlikely to be captured by bulk tumor-level endpoints alone; instead, approaches such as single-cell transcriptomics, spatial profiling, and functional organoid assays are better suited to resolve these cell-state-specific effects. Patient-derived organoids, including CCSC-enriched and matched normal crypt organoids, provide a particularly attractive platform to model differential pathway dependencies, test CCSC-directed combinations, and compare toxicity profiles in vitro before clinical deployment [250,251]. Coupling these organoid models with CRISPR-based perturbation screens could systematically identify genes and pathways that are essential for CCSC fitness but dispensable for normal intestinal stem cells, thereby refining the target space for safer CCSC-directed therapies.

### 11.5. Translational Barriers and Lessons from Completed and Terminated Trials

The high proportion of terminated, withdrawn, and unknown-status trials in the CCSC/stemness dataset highlights persistent translational barriers. Among the 21 interventional CRC trials retrieved, 5 were terminated, 1 was withdrawn, and 2 remained of unknown status, and the documented reasons for early closure illustrate the field’s most pressing operational challenges. Slow or insufficient accrual was the most frequently cited cause. The metformin/CD133 study (NCT01440127) and the pre-operative decitabine study (NCT01882660) were both terminated because patient inclusion was slow and could not reach the target within the study period. The mithramycin trial (NCT02859415), which evaluated a CSC-signaling inhibitor across CCSC-relevant tumor types, was similarly terminated due to slow or insufficient accrual. Beyond accrual, the BNC101 anti-LGR5 antibody study (NCT02726334) completed its monotherapy arm but was terminated before its combination arm could proceed due to sponsor decision, and a separate rectal cancer stem cell marker study (NCT00960427) was withdrawn before enrollment because no surgeon was available to perform the required gastroscopy. The napabucasin (GB201) phase 3 trial (NCT03522649) has not posted results despite its 668-patient enrollment target, reflecting an additional translational gap in which completed studies do not contribute findings to subsequent design decisions. This is not an isolated instance: results have been posted for only a small minority of the 21 retrieved trials, including none for the CSC vaccine study (NCT02176746) a decade after its completion. These patterns indicate that CCSC trials face structurally higher accrual barriers than conventional oncology trials, driven by mandatory molecular prescreening, fresh tissue collection requirements, restrictive eligibility criteria, and dependence on specialized surgical and laboratory infrastructure. Future CCSC-directed studies will benefit from multi-institutional networks with centralized molecular testing, broader use of archived tissue and minimally invasive biomarkers, adaptive trial designs that accommodate molecularly defined subgroups, and pre-specified contingency plans for sponsor-driven discontinuation.

Beyond these operational constraints, the completed trials yield three further lessons. First, target selection has outpaced patient selection. The only trial powered for overall survival, CanStem303C (NCT02753127), enrolled an essentially unselected population and failed, whereas the contemporary marker-directed programs (NCT05759728, NCT03526835) require documented LGR5 expression at screening. This shift toward enrichment is almost certainly necessary, but no CCSC marker has yet been prospectively validated as a predictive biomarker, and enrichment simultaneously compounds the accrual barriers described above. Second, pharmacodynamic endpoints have proven measurable but not decision-grade. CD133 expression, Wnt target gene panels, and single-cell LGR5^+^ signatures can all be quantified in tissue, yet none has been prospectively linked to a clinical outcome, and CD133 remains confounded by its expression on normal regenerative progenitors. Third, pathway redundancy and tumor-cell plasticity remain the dominant biological obstacle: both STAT3-directed (napabucasin) and Notch/DLL4-directed (demcizumab, OMP-305B83) programs failed to advance despite strong preclinical rationale, arguing that CCSC-directed agents should be developed within combination regimens paired with cytotoxic or immune backbones rather than as monotherapy. Taken together with the accrual and reporting gaps, these findings suggest that future CCSC trials should prespecify stem-cell-resolved biomarkers as hierarchically ordered secondary endpoints with predefined assay cutoffs, and commit to timely results posting, so that mechanistic failure can be distinguished from operational failure.

## 12. Conclusions

CCSCs are increasingly recognized as central drivers of tumor initiation, metastatic spread, and resistance to therapy. At the same time, current evidence makes clear that CCSCs represent a heterogeneous set of stem-like states rather than a single, static entity. Surface markers such as CD133, CD44, ALDH1, EpCAM, CD166, and LGR5 enrich for putative CCSCs. However, functional validation consistently shows that marker-defined populations only partially overlap, and that no individual marker provides a universally specific or clinically sufficient definition of CCSCs. CCSC behavior is further shaped by metabolic reprogramming, immunosuppressive niche interactions, and extracellular-vesicle-mediated communication with stromal and immune compartments. These processes collectively promote immune evasion, metastatic niche formation, and treatment resistance. CCSC-directed insights are beginning to inform clinical strategies that include LGR5-directed cellular and bispecific platforms, CSC-focused immunotherapies, stemness-targeting agents, and stem cell-resolved single-cell endpoints. Current outcomes with these approaches remain early and largely exploratory, and they have not yet overcome the high rates of recurrence and therapy resistance seen in advanced CRC. Taken together, these insights suggest that future CCSC-directed approaches in CRC will need to rely on integrated assessments of several factors. These factors include marker expression, functional tumor-initiating capacity, niche and immune context, metabolic state, and emerging precision-oncology tools such as patient-derived organoids and CRISPR-based functional screens. As a result, there is a clear need to move from proof-of-concept CCSC-oriented interventions toward rigorously tested combination regimens that simultaneously target CCSC-intrinsic stemness programs and the microenvironmental drivers of plasticity and resistance. If successfully implemented, such strategies can open a realistic path toward more durable control of advanced CRC and measurable improvements in survival and long-term disease control.

## Figures and Tables

**Table 1:** Overview of colorectal cancer stem cell biomarkers: methods, evidence, and clinical relevance.

Marker	Typical Detection Method	Key Functional Evidence for Tumor-Initiating Capacity	Human Prognostic Evidence	Major Limitations	Current Clinical Applicability
CD133	Immunohistochemistry or flow cytometry using AC133 epitope-specific antibodies.	Early xenograft studies showed CD133^+^ cells are enriched for clonogenic, tumor-initiating activity, with serial transplantation and recapitulation of parental tumor heterogeneity.	Frequently associated with advanced stage and reduced survival in CRC cohorts; proposed as an adverse prognostic marker.	Ubiquitous expression on differentiated epithelium; CD133^−^ cells can also initiate tumors, and CD133 repression has limited functional impact, undermining specificity.	Not used as a standalone clinical test; mainly research-level marker and sometimes included in exploratory prognostic panels.
LGR5	Immunohistochemistry or in situ hybridization; lineage-tracing or reporter alleles in experimental models.	Identifies cycling crypt base stem cells; Lgr5^+^ intestinal cells in mice are highly tumorigenic compared with differentiated cells, and Lgr5^+^ subpopulations in CRC are enriched for CSC-like traits.	Overexpression generally associates with aggressive behavior and reduced survival, though some cohorts show context-dependent or even favorable associations.	Reliable clinical-grade antibodies and standardized scoring are still evolving; expression patterns are context-dependent and may reflect plastic CCSC states rather than a fixed compartment.	Not yet implemented in routine pathology; used in research to study stemness and as a candidate prognostic biomarker and therapeutic target.
CD44	Immunohistochemistry or flow cytometry using pan-CD44 or variant-specific antibodies.	CD44^+^ colon cancer cells show high tumorigenicity and sphere formation; knockdown reduces clonogenicity and xenograft growth.	Elevated CD44 (including CD44v6) often correlates with metastasis, invasion, and poorer outcomes; loss of adhesion-related CD44 at invasive fronts can also mark progression.	Broad expression on stromal and inflammatory cells; splice-variant heterogeneity complicates standardized detection of functionally relevant isoforms.	No routine diagnostic use; considered a promising therapeutic and prognostic target but currently confined to research and biomarker discovery studies.
ALDH1	Enzymatic activity assays (e.g., ALDEFLUOR) and immunohistochemistry for ALDH1 protein.	ALDH^high^ cells from colon cancers are enriched for tumor-initiating capacity in limiting dilution xenografts, and ALDH1^+^ crypt cells expand during adenoma-carcinoma progression.	High ALDH1 expression often correlates with advanced stage and poor survival, supporting its role as a stemness-linked adverse prognostic marker.	Functional assays can capture heterogeneous ALDH^high^ populations including non-CSCs; assay conditions, gating, and cut-offs vary, limiting comparability.	Not a routine clinical test; widely used in research as a functional CSC readout and candidate prognostic biomarker.
EpCAM	Immunohistochemistry and flow cytometry; often combined with other markers (e.g., EpCAM^high^/CD44^+^).	EpCAM^high^/CD44^+^ and EpCAM^high^/CD44^+^/CD166^+^ fractions are enriched for tumor-initiating cells that can regenerate heterogeneous tumors in xenografts.	Altered EpCAM expression (often high in bulk tumor but lost at invasive front) associates with invasion, nodal involvement, and survival differences in large CRC cohorts.	Widely expressed on normal epithelium; not CCSC-specific, and membranous loss can mark invasion, making interpretation context-dependent	Used as part of multiparameter panels and circulating tumor cell enrichment platforms; not applied as a CCSC-specific clinical biomarker on its own.

**Table 2:** Clinical trials relevant CCSC biology and stemness-associated therapeutic strategies in CRC window-of-opportunity studies, prevention studies, and related translational studies (ClinicalTrials.gov; database cutoff 30 July 2026).

Trial/Identifier	Tumor Type	Intervention	Phase	Primary CCSC Target/Strategy	Key Outcome	Status
**Direct CCSC marker-targeted therapies**						
NCT02176746	Metastatic colorectal adenocarcinoma	Cancer stem cell vaccine targeting ALDH^+^ CSC-restricted antigens	Phase 1/2	ALDH^+^ CCSCs	Adverse events; not reported	Completed (2015)
NCT05759728	Metastatic colorectal cancer	CNA3103 autologous CAR-T cells	Phase 1/2a	LGR5	Safety; best overall response	Recruiting;
NCT03526835	Advanced solid tumors including CRC	MCLA-158 (petosemtamab) ± pembrolizumab/FOLFIRI/FOLFOX	Phase 1/2	EGFR × LGR5	DLTs and ORR; interim metastatic CRC data reported responses in 80% of first-line and 62% of second-line patients with FOLFOX/FOLFIRI, and 10% with monotherapy in later lines, with no new safety signals [228]	Recruiting;
NCT02726334	Metastatic colorectal cancer	BNC101 monoclonal antibody ± FOLFIRI	Phase 1	LGR5	MTD in monotherapy escalation; combination arm not evaluated	Terminated (2018); 22 participants
NCT02541370	Multiple solid tumors including CRC	CART133	Phase 1/2	CD133	Safety; no CRC-specific outcomes reported [229]	Completed (2019)
**Stemness pathway-targeted therapies**						
NCT02753127	Previously treated metastatic CRC	Napabucasin + FOLFIRI ± bevacizumab	Phase 3	STAT3-driven stemness	Overall survival; no improvement over FOLFIRI alone [230]	Completed (2021)
NCT03522649	Previously treated metastatic CRC	Napabucasin + FOLFIRI	Phase 3	STAT3-driven stemness	Overall survival; not reported	Unknown
NCT01440127	Colon cancer	Metformin	Phase 1	CD133 modulation/AMPK	Tumor CD133 expression; not reported (early termination)	Terminated (2012)
NCT01882660	Colon cancer	Decitabine	NA	Wnt/β-catenin	Wnt target genes; no consistent induction [231]	Terminated (2018)
NCT02077777	Colorectal cancer	Mesalazine	Phase 2	β-catenin signaling	Gene expression- μ-protocadherin and β-catenin target gene expression; no substantial between-group difference	Completed (2016)
NCT03035253	Metastatic colorectal cancer	OMP-305B83 + FOLFIRI/FOLFOX	Phase 1	DLL4/VEGF (Notch)	Incidence of dose-limiting toxicities; not reported	Terminated (2018)
NCT01189942	Metastatic colorectal cancer	Demcizumab (OMP-21M18) + FOLFIRI	Phase 1	DLL4 (Notch)	MTD of demcizumab with FOLFIRI; not reported	Completed (2011)
NCT02859415	Multiple solid tumors including CRC	Mithramycin	Phase 1/2	Stemness signaling	MTD not determined; no objective responses	Terminated (2021)
NCT05630794	Adenomatous polyps/CRC/FAP	ONC201 (dordaviprone)	Phase 1	CD133, CD44, ALDH, LGR5	Safety	Recruiting
**Biomarker and window-of-opportunity studies**						
NCT01483001	Lung, breast, CRC and others	Cancer stem-cell chemosensitivity assay	Phase 2	CD133 biomarker	Assay feasibility met; predictive validity not established [232]	Completed (2013)
NCT03085992	Resectable rectal cancer	FOLFOXIRI + bevacizumab	Phase 2	CD133 biomarker	Two-year disease-free survival [233]	Completed (2018)
NCT00960427	Stage II/III rectal cancer	Chemoradiotherapy with stem-cell marker analysis	Phase 1	Stem-cell biomarkers	CCSC marker expression and correlation with disease-free survival; never assessed	Withdrawn (2010)
**Prevention studies**						
NCT05056896	Colorectal cancer prevention	Aspirin vs. placebo (ASPIRED-XT)	Early Phase 1	LGR5^+^ intestinal stem-cell markers	Change in LGR5⁺ stem cells; age-dependent effect observed [234]	Active
**Other CCSC-related translational studies**						
NCT00601549	Advanced rectal cancer	Laparoscopic vs. open surgery after CCRT	Phase 3	Stem-cell biology correlation	Oncologic outcomes and functional recovery; not reported	Unknown

DLT: dose-limiting toxicity; ORR: objective response rate; MTD: maximum tolerated dose.

## Data Availability

No new data were created or analyzed in this study. Data sharing is not applicable to this article.
